# Single‐Layered MoS_2_ Fabricated by Charge‐Driven Interlayer Expansion for Superior Lithium/Sodium/Potassium‐Ion‐Battery Anodes

**DOI:** 10.1002/advs.202207234

**Published:** 2023-03-22

**Authors:** Zhenwei Li, Meisheng Han, Yuanbo Zhang, Fu Yuan, Ying Fu, Jie Yu

**Affiliations:** ^1^ Songshan Lake Materials Laboratory Dongguan Guangdong 523808 P. R. China; ^2^ Guangdong Provincial Key Laboratory of Semiconductor Optoelectronic Materials and Intelligent Photonic Systems Shenzhen Engineering Lab for Supercapacitor Materials School of Material Science and Engineering Harbin Institute of Technology Shenzhen University Town Shenzhen 518055 P. R. China; ^3^ Department of Mechanical and Energy Engineering Southern University of Science and Technology Shenzhen 518055 P. R. China

**Keywords:** charge‐driven interlayer expansion, Co doping, Li/Na/K‐ion batteries, single‐layered MoS_2_, spin‐polarized surface capacitance

## Abstract

Single‐layered MoS_2_ is a promising anode material for lithium‐ion batteries (LIBs), sodium‐ion batteries (SIBs), and potassium‐ion batteries (PIBs) due to its high capacity and isotropic ion transport paths. However, the low intrinsic conductivity and easy‐agglomerated feature hamper its applications. Here, a charge‐driven interlayer expansion strategy that Co^2+^ replaces Mo^4+^ in the doping form to endow MoS_2_ layers with negative charges, thus inducing electrostatic repulsion, together with the insertion of gaseous groups, to drive interlayer expansion which once breaks the confinement of interlayer van der Waals force, single‐layered MoS_2_ is obtained and uniformly dispersed into carbon matrix arising from the transformation of carbonaceous gaseous groups under high vapor pressure, is proposed. Co atom doping helps enhance the intrinsic conductivity of single‐layered MoS_2_. Carbon matrix effectively prevents agglomeration of single‐layered MoS_2_. The doped Co atoms can be fully transformed into ultrasmall Co nanoparticles during conversion reaction, which enables strong spin‐polarized surface capacitance and thus significantly boosts ion transport and storage. Consequently, the prepared material delivers superb Li/Na/K‐ion storage performances, which are best in the reported MoS_2_‐based anodes. The proposed charge‐driven interlayer expansion strategy provides a novel perspective for preparing single‐layered MoS_2,_ which shows huge potential for energy storage.

## Graphical abstract

Single‐layered MoS_2_ has great potential in energy storage but suffers from low intrinsic conductivity and easy‐agglomerated feature. Here, a charge‐driven interlayer expansion strategy is proposed. The interlayer electrostatic repulsion induced by Co doping breaks the interlayer van der Waals force to fabricate Co‐doped single‐layered MoS_2_, which uniformly disperses in carbon matrix and shows superior lithium/sodium/potassium‐ion transport and storage capability.

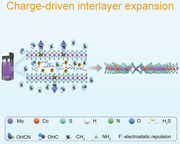

## 1 Introduction

Rechargeable lithium‐ion batteries (LIBs), sodium‐ion batteries (SIBs), and potassium‐ion batteries (PIBs) have been extensively studied and partially commercialized.^[^ ^1^, ^2^, ^3^ ^]^ As the most popular anode material, graphite has shown huge success for LIBs over the past decade. However, graphite suffers from low specific capacity (372 mAh g^−1^) and unsatisfactory rate capability mainly caused by anisotropic lithium‐ion storage paths and small interlayer distance (0.334 nm).^[^ ^4^ ^]^ Graphite is becoming increasingly difficult to cope with the growing demand for high energy density and fast charging capability in LIBs. Meanwhile, graphite is not suitable as anode material for SIBs owing to the thermodynamic instability of the binary Na‐intercalated graphite compounds,^[^ ^5^, ^6^ ^]^ and also exhibits low capacity of 279 mAh g^−1^ and poor rate performance for PIBs.^[^ ^7^ ^]^ In this context, developing a universal high‐performance anode material for LIBs, SIBs, and PIBs is of practical importance.

MoS_2_ is a potential anode material for LIBs, SIBs, and PIBs because of its high theoretical capacity (670 mAh g^−1^) and unique layered structure with a relatively large interlayer space of 0.62 nm.^[^ ^8^ ^]^ However, there are still some obstacles to be addressed in developing high‐performance MoS_2_ electrodes. The main challenges arise from its unsatisfactory intrinsic electrical conductivity (EC), large ion diffusion barrier, and large volume change upon cycling. These issues result in poor rate capability and cycle stability and thus severely restrict its application in LIBs, SIBs, and PIBs.^[^ ^9^, ^10^, ^11^ ^]^

Many different strategies including enlarging interlayer distance of MoS_2_,^[^ ^12^, ^13^, ^14^ ^]^ constructing MoS_2_/graphene interoverlapped structure,^[^ ^11^, ^15^ ^]^ doping heteroatom,^[^ ^16^, ^17^ ^]^ fabricating few‐layered or even single‐layered MoS_2_,^[^ ^18^, ^19^ ^]^ and compositing with carbon materials^[^ ^20^ ^]^ have been implemented to tackle with the above‐mentioned tricky problems. Although few‐layered MoS_2_ have larger interlayer spaces and are cheap in production, the adjacent layers form barriers to ion transport, resulting in much longer and more tortuous ion transport paths. On the contrary, the single‐layered MoS_2_ is completely exposed and easily accessible to the ions transported from all directions, providing short and easy ion transport paths.^[^ ^21^, ^22^ ^]^ In addition, the two surfaces of the single‐layered MoS_2_ are capable of storing ions, increasing the ion storage capacity. Previous reports also showed that the electrochemical properties of single‐layered MoS_2_ are obviously better than few‐layered MoS_2_,^[^ ^11^, ^23^ ^]^ suggesting the great potential of single‐layered MoS_2_ on LIB/SIB/PIB applications. Many methods have been reported to prepare the single‐layered MoS_2_, e.g., one‐pot colloidal wet‐chemical approach,^[^ ^24^ ^]^ mechanical/chemical exfoliation,^[^ ^25^, ^26^, ^27^ ^]^ and chemical vapor deposition.^[^ ^28^ ^]^ Especially, the methods of using minerals as precursors based on top‐down exfoliation process show great potential in the mass production of single‐layered MoS_2_ and other 2D materials.^[^ ^29^, ^30^, ^31^ ^]^ Notwithstanding the outcomes in preparing single‐layered MoS_2_, its poor intrinsic EC and serious restacking/agglomeration problems severely inhibit its application in batteries. To circumvent these difficulties, various techniques including solvothermal synthesis,^[^ ^32^ ^]^ hydrothermal method,^[^ ^33^ ^]^ and CTAB‐assisted synthesis^[^ ^34^ ^]^ have been involved to construct the sandwich structure of single‐layered MoS_2_/graphene, which effectively improves EC of the electrode and avoids agglomeration of the single‐layered MoS_2_ and thereby enhanced electrochemical performances. However, such a sandwich structure reintroduces the interlayer ion diffusion barrier. Although the interlayer space of the adjacent MoS_2_ layers in the sandwich structure is enlarged (≈0.96 to ≈1.15 nm) compared to the theoretical value (0.62 nm), the presence of graphene between adjacent MoS_2_ layers makes the actual interlayer distance (from MoS_2_ to graphene) available for ion transport to be only ≈0.53 nm, which is even smaller than that of bulk MoS_2_. This inevitably sets up barriers for the transport of Li^+^ in the interlayer, let alone Na^+^ and K^+^ with a larger radius. Given this situation, further efforts such as electrospinning,^[^ ^23^ ^]^ dual‐template method,^[^ ^35^ ^]^ and emulsion‐templated solvothermal method,^[^ ^36^ ^]^ have been carried out to synthesize composites with uniformly dispersed single‐layered MoS_2_ in carbon matrix. This strategy eliminates the interlayer ion diffusion energy barrier while suppressing the agglomeration of single‐layered MoS_2_. The corresponding electrochemical performances are also greatly improved based on the advantages of uniformly dispersed single‐layered MoS_2_. Unfortunately, the intrinsic electrical conductivity of MoS_2_ is not increased, far from being sufficient to perform fast charge storage, thus causing the insufficient release of the huge potential of single‐layered MoS_2_ in the capacity and rate performances. Hence, it is of very importance to develop a novel route to synthesize single‐layered MoS_2_ with enhanced intrinsic electrical conductivity and uniformly dispersed in carbon matrix to achieve superior Li/Na/K‐ion storage performances, which will certainly boost the application pace of MoS_2_ materials in LIBs, SIBs, and PIBs.

Heteroatom doping can enlarge the interlayer distance of layered transition metal sulfides due to the electrostatic repulsion between the adjacent layers caused by atomic substitution during the doping process, showing high potential in MoS_2_‐based electrode material.^[^ ^17^ ^]^ Recently, Li et al. demonstrated the presence of strong spin‐polarized surface capacitance on Co nanoparticles in LIBs system. Specifically, the CoO electrode can generate ultrasmall Co nanoparticles during conversion reactions, and numerous spin‐polarized electrons are injected into Co nanoparticles while lithium ions are stored in Li_2_O matrix, causing a spin capacitance.^[^ ^37^ ^]^ It is reasonable to believe that a strategy that introduces Co atom into MoS_2_ lattices in the form of doping cannot only induce the charge‐driven interlayer expansion effect to obtain the single‐layered MoS_2_, but also bring surface capacitance effect during conversion reactions, thus boosting ion transport and storage.

Herein, we propose a novel charge‐driven interlayer expansion strategy to prepare single‐layered MoS_2_ with enhanced intrinsic EC in carbon matrix based on Co doping. Co^2+^ replaces Mo^4+^ of MoS_2_ lattice to complete the doping process, which results in MoS_2_ layers with negative charges to induce the interlayer electrostatic repulsion, together with the insertion of gaseous groups, to drive interlayer expansion. When the electrostatic repulsion is large enough to break the limitation of interlayer van der Waals forces, the Co‐doped single‐layered MoS_2_ is obtained and uniformly dispersed in N, O co‐doped carbon matrix (Co‐SLMoS_2_/NOC). Utilizing Co‐SLMoS_2_/NOC as anode material for LIBs, SIBs, and PIBs holds the following advantages: 1) the fully exposed single‐layered MoS_2_ basal planes can perform fast charge transport due to the disappearance of interlayer ion transport barrier; 2) Co doping hugely enhances the intrinsic EC of single‐layered MoS_2_, thereby significantly accelerating electrons transfer; 3) Co‐SLMoS_2_/NOC has a maximization of contact area between single‐layered MoS_2_ and carbon matrix to fully improve the electrical conductivity of single‐layered MoS_2_, thus accelerating the transfer rate of charges; 4) the doped Co atoms in Co‐SLMoS_2_/NOC can transform into ultrasmall Co nanoparticles (≈2 nm) during conversion reaction, which can generate strong spin‐polarized surface capacitance to enhance ion transport and storage. As a result, the Co‐SLMoS_2_/NOC delivers the best comprehensive electrochemical performances in terms of capacity, cyclability, and rate performances compared with the recently reported MoS_2_‐based anode materials for LIBs, SIBs, and PIBs.

## 2 Results and Discussion

### 2.1 Fabrication of Samples

**Figure 1:**
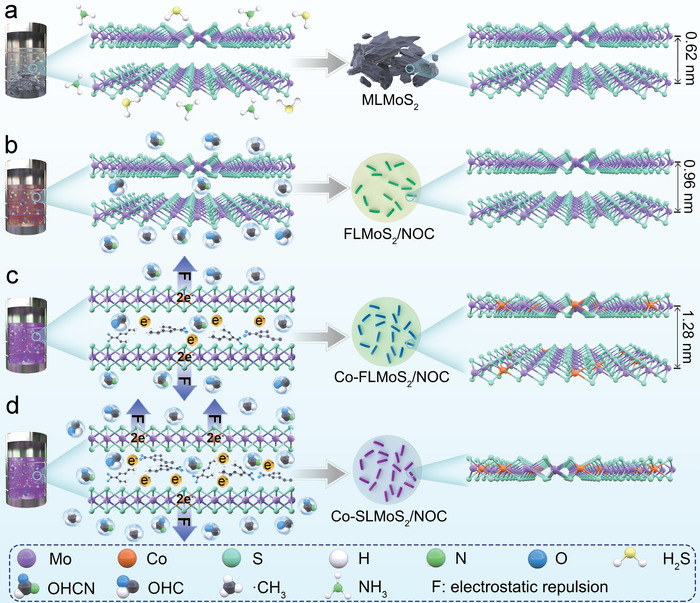
Schematic diagram of the synthesis process of a) MLMoS_2_, b) FLMoS_2_/NOC, c) Co‐FLMoS_2_/NOC, and d) Co‐SLMoS_2_/NOC samples.

**Figure 2:**
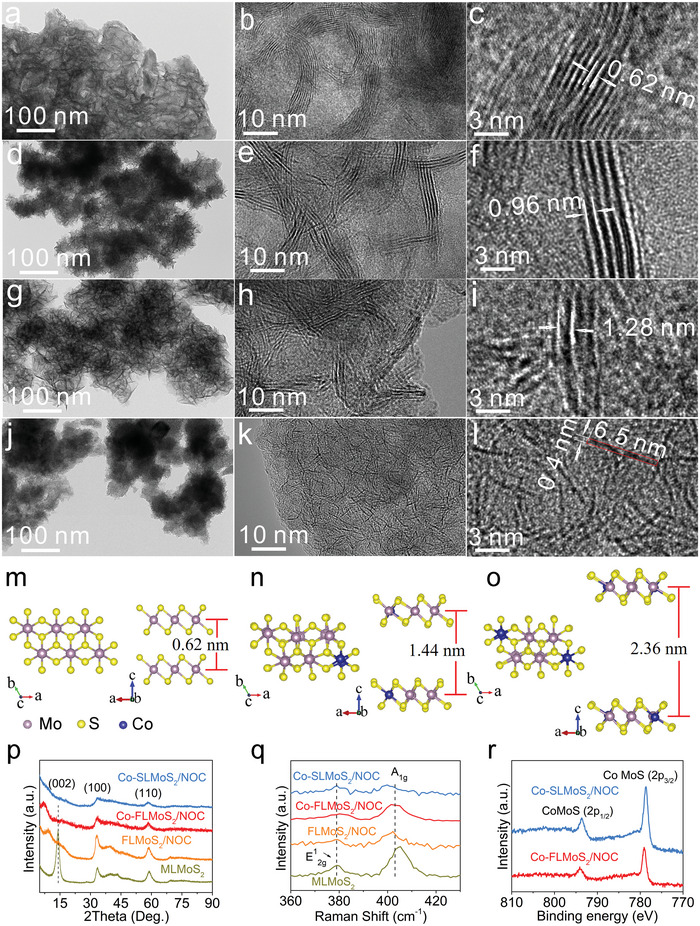
a–l) TEM and HRTEM images of a–c) MLMoS_2_, d–f) FLMoS_2_/NOC, g–i) Co‐FLMoS_2_/NOC, and j–l) Co‐SLMoS_2_/NOC. The DFT calculated interlayer distance of MoS_2_: m) without Co doping, n,o) Co doping with a Co/Mo atomic ratio of n) 1/5 and o) 1/2 (meanwhile introducing the corresponding number of negative charges). p) XRD patterns, q) Raman spectra, and r) high‐resolution XPS spectra of Co of the prepared samples.

The samples are prepared by pyrolysis of the precursor solution containing distinct amounts of cobalt naphthenate, (NH_4_)_2_MoS_4_, and *N*, *N*‐dimethylformamide (DMF) through a facile one‐step pressure‐induced vapor synthetic route (**Figure** **1**, detailed experimental content in Supporting Information). The corresponding microstructure characterizations of the prepared samples are shown in **Figure** **2**. As shown in Figure 1a, (NH_4_)_2_MoS_4_ thermally decomposes to form multilayered MoS_2_ (MLMoS_2_, ∼10 layers) with a typical interlayer space of 0.62 nm, which is confirmed by high‐resolution transmission electron microscopy (HRTEM) observation (Figure 2b,c). After introducing DMF (Figure 1b), vast gaseous groups (OHCN, OHC, ·CH_3_, etc.) caused by the pyrolysis of DMF insert the MoS_2_ interlayer, which induces the reduction of MoS_2_ layers (3–5 layers, Figure 2d,e) and the enlargement of interlayer distance (0.96 nm, Figure 2f). Meanwhile, the pyrolysis products surrounding the few‐layered MoS_2_ can be transformed into N, O co‐doped carbon matrix under large vapor pressure and annealing to obtain FLMoS_2_/NOC sample. Cobalt naphthenate, an organic ionic compound ( [(C_5_H_9_)(CH_2_)_n_COO]_2_Co containing one positively charged Co^2+^ and two negatively charged (C_5_H_9_)(CH_2_)_n_COO^−^), is further introduced into the system as the dopant to achieve Co doping for MoS_2_ (Figure 1c). It is worth noting that Co^2+^ replaces Mo^4+^ of the MoS_2_ lattice to make MoS_2_ layers with negative charges and leave negatively charged gaseous groups (carboxylic anions) in the interlayer, thus inducing interlayer electrostatic repulsion to achieve the charge‐driven interlayer expansion for MoS_2_. As can be seen in Figures 2g‐i, a further increased interlayer distance of 1.28 nm for few‐layered MoS_2_ can be obtained when introducing a small amount of cobalt naphthenate (named as Co‐FLMoS_2_/NOC). Continue to increase the amount of cobalt naphthenate can increase the number of the negative charges to bring stronger interlayer electrostatic repulsion (Figure 1d), which is evidenced by the disappeared few‐layered MoS_2_ and the formed numerous single‐layered MoS_2_ (Figures 2j,k), thus obtaining Co‐SLMoS_2_/NOC sample. This means that the increased interlayer electrostatic repulsion far surpasses the interlayer van der Waals interaction of MoS_2_. It is clearly indicated that the present material form with the MoS_2_ layers embedded in the carbon matrix avoids agglomeration of the MoS_2_ layers (Figure 2k). Meanwhile, the single‐layered MoS_2_ is extremely small (a length size of ≈6.5 nm) and thin (a thickness size of ≈0.4 nm, in agreement with the standard distance data of single‐layered MoS_2_ (JCPDS 37–1492); Figure 2l). The lateral dimension of ≈6.5 nm enables extremely short lateral transport length of charges and small local electroactive mass. The latter point is crucial for the spin‐polarized surface capacitance (discussed below). Note that the lateral size decreased from ≈12 nm of Co‐FLMoS_2_/NOC to ≈6.5 nm of Co‐SLMoS_2_/NOC. This may be because 1) the short time for MoS_2_ growth is not enough to obtain the large‐sized MoS_2_ layers in the pressure‐induced vapor synthetic system, and 2) the repulsion of Co‐SLMoS_2_/NOC system is much larger than that of Co‐FLMoS_2_/NOC system during the synthesis process (discussed below), which may inhibit the growth of MoS_2_, thus leading to a smaller size of MoS_2_ in Co‐SLMoS_2_/NOC compared to Co‐FLMoS_2_/NOC. In addition, the structure of MoS_2_ remains intact after Co doping, demonstrating the successful fabrication of Co‐doped single‐layered MoS_2_ in Co‐SLMoS_2_/NOC. The morphology of the synthesized samples is exhibited in Figure S1 (Supporting Information), irregular micron‐scale bulks are obtained for (NH_4_)_2_MoS_4_ and MLMoS_2_. However, FLMoS_2_/NOC, Co‐FLMoS_2_/NOC, and Co‐SLMoS_2_/NOC display a nano‐sized morphology, which can effectively shorten the ion transport length and relieve the stress caused by the volume expansion of the electrode. Besides, the high‐angle annular dark‐field (HAADF) images and the energy‐dispersive X‐ray spectroscopy (EDS) elemental mapping confirm the uniform distribution of all expected elements in FLMoS_2_/NOC (Figure S2, Supporting Information), Co‐FLMoS_2_/NOC (Figure S3, Supporting Information), and Co‐SLMoS_2_/NOC (Figure S4, Supporting Information).

Density functional theory (DFT) calculations are carried out to further evidence the reliability of the charge‐driven interlayer expansion (the detailed computational method exhibited in Supporting Information). Specifically, we investigate the equilibrium interlayer spacing of adjacent MoS_2_ with different additional charge concentrations caused by the Co doping process. For example, when two Co^2+^ replace two Mo^4+^ of adjacent MoS_2_ layers to complete the doping, the two adjacent MoS_2_ layers each carry two negative charges. Meanwhile, the donor (cobalt naphthenate) of these two Co^2+^ provides four negative charges in the interlayer, together with the four negative charges on MoS_2_ layers to leave eight negative charges for the MoS_2_ model. The accumulated negative charges can induce interlayer electrostatic repulsion, leading to the expansion of the interlayer distance of MoS_2_. Note that the simulation of charge amounts by regulating the number of electrons in the model. In Figure 2m, MoS_2_ maintains its typical interlayer distance of 0.62 nm under a vacuum layer thickness of 30 Å. For the model with enlarged interlayer distance (corresponding to Co‐FLMoS_2_/NOC), a Co/Mo atomic ratio of 1:5 is constructed (from XPS analysis results in Table S2, Supporting Information), and the corresponding negative charges are also introduced to the MoS_2_ model (Figure 2n). Note that the interlayer distance of MoS_2_ calculated from the model should be lower than that in Co‐FLMoS_2_/NOC. This is because the interlayer distance of MoS_2_ in Co‐FLMoS_2_/NOC is caused by the combined impetus of the insertion of gaseous groups and the charge‐driven interlayer repulsion rather than the single interlayer repulsion set for the model. However, the calculated interlayer distance (1.44 nm) is slightly larger than in Co‐FLMoS_2_/NOC (1.28 nm) under a similar Co doping amount (based on XPS results below). This may be because of the volume shrinkage arising from the conversion from gas to the solid phase under high vapor phase pressure and the incomplete insertion of negatively charged gaseous groups in the interlayer. Further increasing the number of Co atoms and negative charges in the model (≈1/2, atomic ratio of Co/Mo, Table S2, Supporting Information), a hugely increased interlayer distance of 2.36 nm is obtained (Figure 2o), which signals the formation of single‐layered MoS_2_.^[^ ^38^ ^]^ Importantly, the lattice constants of MoS_2_ in models are well preserved after interlayer expansion and separation. Although there are some differences in the interlayer distance between the calculation in the model and the experimental samples, the variation trend of the interlayer distance keeps consistent under charge driving. This result demonstrates the effectiveness of the charge‐driven interlayer expansion strategy. This expansion method is promising in the mass production of single‐layered MoS_2_, which is dependent on the successful construction of the production equipment. It is expected that this method is of high potential to produce other 2D materials following a similar principle.

Figure 2p presents the X‐ray diffraction (XRD) pattern of the synthesized samples. All samples display the (100) and (110) diffraction peaks of 2H‐MoS_2_ (JCPDS: 37–1492), signaling the successful preparation of MoS_2_ in these samples. The (002) diffraction peak of MLMoS_2_ (14.3°), FLMoS_2_/NOC (9.2°), and Co‐FLMoS_2_/NOC (7.0°) samples can be used to analyze their interlayer distance, and the corresponding values are 0.62, 0.96, and 1.26 nm, respectively (according to the Bragg's Law). No (002) diffraction peak can be detected in Co‐SLMoS_2_/NOC, demonstrating the presence of single‐layered MoS_2_.^[^ ^23^, ^36^ ^]^ These results strongly support that the single‐layered MoS_2_ can be prepared by the charge‐driven interlayer expansion strategy and are consistent with the HRTEM results (Figure 2k,l). In addition, no graphitic peak at ≈25° can be detected, indicating the carbon phase in these samples is amorphous. Figure 2q exhibits the Raman spectra, in which two characteristic peaks at ≈380 and 405 cm^−1^ correspond to the in‐plane vibration (E^1^_2g_) and out‐of‐plane vibration (A_1g_) of MoS_2_, respectively.^[^ ^39^ ^]^ Note that the difference in peak frequency of E^1^_2g_ and A_1g_ can offer layer number information for MoS_2_.^[^ ^40^ ^]^ The frequency differences for FLMoS_2_/NOC and Co‐FLMoS_2_/NOC are 22.4 and 21.9 cm^−1^ (≈3 layers), respectively, as compared with 26.2 cm^−1^ (>5 layers) for MLMoS_2_. The reduction of frequency difference indicates that FLMoS_2_/NOC and Co‐FLMoS_2_/NOC possess a thinner layer structure than MLMoS_2_.^[^ ^40^ ^]^ Especially, the frequency difference of Co‐SLMoS_2_/NOC (20.5 cm^−1^) falls within the range of single‐layered MoS_2_ (20.2–21.2 cm^−1^),^[^ ^40^ ^]^ which further evidence that single‐layered MoS_2_ exists in Co‐SLMoS_2_/NOC and is consistent with HRTEM (Figure 2l) and XRD (Figure 2p) results. In addition, no carbon peaks can be detected in MLMoS_2_, whereas FLMoS_2_/NOC, Co‐FLMoS_2_/NOC, and Co‐SLMoS_2_/NOC show the characteristic peaks of D‐band (≈1339.5 cm^−1^) and G‐band (≈1588.3 cm^−1^) of carbon‐based materials (Figure S5, Supporting Information) with a relatively high *I*_D_/*I*_G_ intensity ratio of ≈0.93 (Table S1, Supporting Information). This indicates rich structural defects in the carbon matrix because of N, O co‐doping, which is conducive to the transport of electrons and ions.^[^ ^41^ ^]^

Moreover, the chemical states of these samples are analyzed by X‐ray photoelectron spectrometer (XPS). From Figure S6 and Table S2 (Supporting Information), it can be seen that the existence of all expected elements of Mo, S in MLMoS_2_, Mo, S, C, N, O in FLMoS_2_/NOC, and Co, Mo, S, C, N, O in Co‐FLMoS_2_/NOC and Co‐SLMoS_2_/NOC. Table S3 (Supporting Information) suggests that Co doping amounts in Co‐FLMoS_2_/NOC and Co‐SLMoS_2_/NOC are 5.42 and 11.30 at.%, respectively, which represents an increased tendency of Co doping amounts with the rise of cobalt naphthenate. In Figure S7a,b (Supporting Information), the peaks of Mo 3d (Mo 3d_5/2_, ≈229 eV; Mo 3d_3/2_, ≈232 eV) and S 2p (S 2p_3/2_, ≈162 eV; S 2p_1/2_, ≈163 eV) are attributed to Mo^4+^ and S^2−^, respectively, again indicating the formation of MoS_2_.^[^ ^42^ ^]^ Two distinct peaks at 778.7 and 793.7 eV correspond to the Co 2p_3/2_ and Co 2p_1/2_ orbitals of CoMoS (Figure 2r), respectively,^[^ ^43^ ^]^ denoting that the Co atoms are doped into MoS_2_ lattices to obtain the CoMoS phase in Co‐FLMoS_2_/NOC and Co‐SLMoS_2_/NOC. In the high‐resolution C 1s, N 1s, and O 1s spectra, peaks of 284.5 eV (C—C), 285.8 eV (C—N/C—O), 288.8 eV (C=O) (Figures S7c and S8, Supporting Information), 397.7–400.6 eV (pyridinic N, pyrrolic N, and graphitic N, Figures S7d and S9, Supporting Information), and 531.8 eV (C=O; Figure S7e, Supporting Information) confirm the successful doping of N and O into the carbon lattice.^[^ ^44^, ^45^ ^]^ The specific N and O doping amounts in the carbon matrix are shown in Table S4 (Supporting Information). The presence of the carbon matrix obviously enhances the electrical conductivity of the samples (1.3 × 10^−3^ S cm^−1^, MLMoS_2_; 4.8 S cm^−1^, FLMoS_2_/NOC; Table S5, Supporting Information), solving the problem of poor electrical conductivity of MoS_2_. Furthermore, elemental analysis (EA) and thermogravimetric analysis (TGA) are carried out to further analyze the composition of samples. Table S6 (Supporting Information) gives the components of these samples and the residues mass of MLMoS_2_, FLMoS_2_/NOC, Co‐FLMoS_2_/NOC, and Co‐SLMoS_2_/NOC after thermal decomposition in air can be calculated to 89.4, 74.3, 69.2, and 65.4 wt.%, respectively, which are consistent with the TGA results (Figure S7f, Supporting Information). Furthermore, we also explore the effect of excessive Co doping on sample structure. As can be seen in Figure S10 (Supporting Information), the sample with excessive Co doping (≈3/4, Co/Mo atomic ratio, XPS result in Figure S10c, Supporting Information) shows a bulk morphology (Figure S10a, Supporting Information). In Figure S10b (Supporting Information), the presence of the (100) and (110) crystal planes and the absence of the (002) crystal plane of MoS_2_ suggest the existence of single‐layered MoS_2_ in the sample. In addition to the peaks of MoS_2_, a series of additional peaks at 29.9, 31.3, 39.5, 47.6, and 51.9° can be detected, which correspond to the (311), (222), (331), (511), and (440) crystal planes of Co_3_S_4_ (JCPDS: 02–1338), respectively. This confirms that excessive Co doping can induce the formation of Co_3_S_4_. Meanwhile, the two additional peaks at 782.5 and 797.4 eV related to Co_3_S_4_ for the XPS spectra further confirm the formation of Co_3_S_4_ (Figure S10d).^[^ ^46^ ^]^ Therefore, it is believed that the optimal value of Co/Mo atomic ratio for the preparation of single‐layered MoS_2_ in this system is ≈1:2.

### 2.2 Electrochemical Characterizations for LIBs

**Figure 3:**
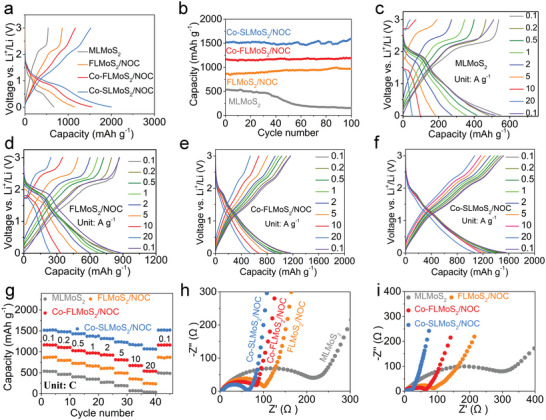
a) First charge and discharge profiles, b) cycling performance at 0.1 A g^−1^, c–f) charge/discharge profiles at different current densities, g) rate curves, and h,i) Nyquist plots before cycling (h) and after testing rate performances (i) of the prepared samples.

**Figure 4:**
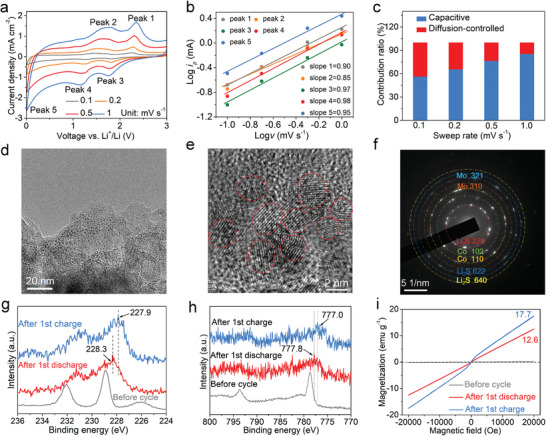
a) CV curves at different sweep rates, b) Log *i_p_* against Log *v* at peaks 1–5, and c) the percentages of pseudocapacitive contribution at different sweep rates of Co‐SLMoS_2_/NOC. d) TEM image, e) HRTEM image, and f) SAED pattern of Co‐SLMoS_2_/NOC after discharging to 0.01 V. Ex situ XPS spectra of Mo 3d (g) and Co 2p (h). i) Ex situ magnetic hysteresis loops of Co‐SLMoS_2_/NOC.

The electrochemical data including the first charge/discharge curves at 0.1 A g^−1^ (**Figure** **3**a), cycling curves at 0.1 A g^−1^ (Figure 3b), and rate capability (Figures 3c‐g) of these samples are tested. As revealed, MLMoS_2_ shows quite poor first charge capacity (533.6 mAh g^−1^), cycle stability (28.4% capacity retention after 100 cycles), and rate capability (37.2 mAh g^−1^ at 20 A g^−1^) because of its micron‐sized bulk feature and the lack of carbon matrix, consistent with the previous report.^[^ ^33^ ^]^ After compounding few‐layered MoS_2_ with carbon matrix, FLMoS_2_/NOC exhibits obvious enhancement in first charge capacity (865.2 mAh g^−1^), cycle stability (112.0% capacity retention after 100 cycles), and rate capability (241.5 mAh g^−1^ at 20 A g^−1^), which are much higher than that of MLMoS_2_. The increased capacity during cycling may be ascribed to the formation of gel‐like polymeric layer, and the increase of interfaces with increasing the cycle number, which can act as active storage sites of lithium ion.^[^ ^16^, ^47^ ^]^ The increase of active interfaces may arise from 1) the appearance of more exposed surfaces as the activation of FLMoS_2_/NOC electrode upon cycling, 2) the redistribution of sulfur species, 3) the formation of ultrasmall Mo nanoparticles during conversion reaction (discussed below). Further introducing Co doping, a better improvement in initial charge capacity (1165.6 mAh g^−1^) and rate capability (536.2 mAh g^−1^ at 20 A g^−1^) are achieved for Co‐FLMoS_2_/NOC due in large part to the strong spin‐polarized surface capacitance effect (confirmed in **Figure** **4**) caused by Co doping and large interlayer distance. Remarkably, Co‐SLMoS_2_/NOC exhibits unprecedented electrochemical performances, far superior to Co‐FLMoS_2_/NOC, FLMoS_2_/NOC, and MLMoS_2_. Specifically, Co‐SLMoS_2_/NOC shows an ultrahigh first charge capacity of 1520.1 mAh g^−1^ with an initial Coulombic efficiency (CE) of 76.3% (Figure 3a), and a high reversible capacity of 1596.2 mAh g^−1^ after 100 cycles (Figure 3b). When gradually increasing the current density from 0.1 to 20 A g^−1^, the charge/discharge profiles at each current density remain stable and deliver a superior rate capacity of 1068.2 mAh g^−1^ at 20 A g^−1^ (≈70.3% capacity retention relative to the capacity obtained at 0.1 A g^−1^). Such superior electrochemical performances of Co‐SLMoS_2_/NOC can be attributed to 1) its vanishing interlayer ion diffusion barrier, more efficient open ion diffusion mode, lower charge transfer resistance (*R*_ct_; Figure 3h,i), higher EC (Table S5, Supporting Information), and bigger specific surface area (Figure S11, Supporting Information) facilitate the ion transport on the MoS_2_ plane, and 2) stronger spin‐polarized surface capacitance is induced due to the formation of smaller Co nanoparticles (discussed below) during the conversion reaction, which hugely boosts ion transport and storage. We point out in passing that the first charge capacity of Co‐doped single‐layered MoS_2_ can reach 1775.0 mAh g^−1^ when removing the contribution of the N, O co‐doped carbon matrix in Co‐SLMoS_2_/NOC (detailed discussion in Figures S12 and S13, Supporting Information). So high capacity of Co‐doped single‐layered MoS_2_ is unprecedented, fully demonstrating the sufficient release of the huge potential of single‐layered MoS_2_ in the capacity by using the charge‐driven interlayer expansion strategy. Remarkably, the Co‐SLMoS_2_/NOC has an ultralow volume swelling of 10.3% in electrode thickness, while the MLMoS_2_ exhibits a thickness change of 82.5% after 100 cycles (Figure S14, Supporting Information), confirming that the structural superiority of Co‐SLMoS_2_/NOC as LIB anode.

### 2.3 Study on the Kinetics and Behavior of Li‐Ion Storage in Co‐SLMoS_2_/NOC

To explain the reason of the excellent rate capability and high capacity of Co‐SLMoS_2_/NOC, electrochemical kinetic studies and Li‐ion storage behavior in Co‐SLMoS_2_/NOC are investigated. Figure 4a displays the cyclic voltammetry (CV) profiles (0.1–1.0 mV s^−1^, sweep rates). The capacitive effect of the Co‐SLMoS_2_/NOC electrode can be calculated based on Equation (1):^[^ ^48^ ^]^

(1) $$i\;=\;av^{b}$$

where *i* is the current density, *v* is the scan rate, and a and b are empirical constants. When the b‐value is close to 1, the cell system is mainly controlled by the capacitance, and when the b‐value approaches 0.5, ion diffusion‐controlled process dominates. In Figure 4b, the b‐values of peaks 1–5 in both cathodic and anodic processes for Co‐SLMoS_2_/NOC are 0.90, 0.85, 0.97, 0.98, and 0.95, respectively. This suggests that Li‐ion storage dynamics are mainly controlled by the capacitive process. Moreover, the capacitive contribution can be further quantified according to Equation (2).^[^ ^49^ ^]^

(2) $$i\;\left(V\right)=k_{1}\;v+k_{2}v^{1/2}$$

where *i(V)*, *k_1_v*, and *k_2_v^1/2^* represent the total current at a fixed potential, the pseudocapacitance behavior, and the diffusion‐controlled process, respectively. With the increase of the sweep rate from 0.1 to 1.0 mV s^−1^, the capacitive contribution enlarges from 56.3% to 85.3% (Figure 4c), indicating that capacitive behavior is essential to the high‐rate capability. In addition, Co‐SLMoS_2_/NOC also shows huge advantages in capacitive contribution when compared with MLMoS_2_ (4.3% to 14.1%, Figure S15, Supporting Information), FLMoS_2_/NOC (24.2% to 46.4%, Figure S16, Supporting Information), and Co‐FLMoS_2_/NOC (41.3% to 67.7%, Figure S17, Supporting Information), further evidence that capacitive behavior boosts Li‐ion transport and storage in Co‐SLMoS_2_/NOC. The Li‐ion diffusion coefficient (*D*_Li_^+^) is calculated to confirm the fast Li‐ion transport dynamics according to Equation (3).^[^ ^50^, ^51^ ^]^

(3) $$i_{p}=\;2.69\times 10^{5}n^{3/2}AD_{Li+}^{\;1/2}C_{Li+}v^{1/2}$$

where *i_p_*, *v*, *A*, *n*, and *C*_Li+_ represent the peak current, the sweep rate, the contact area between the electrolyte and active materials, the number of electrons in the electrochemical reaction, and the Li^+^ bulk concentration (calculated in Supporting information), respectively. According to the linear relationship of *i_p_* versus *v* ^1/2^ of the redox peaks (Figure S18, Supporting Information), the values of *D*_Li+_ for Peaks 1–5 range from 2.65 × 10^−10^ to 1.97 × 10^−9^ cm^2^ s^−1^. So high *D*_Li+_ strongly supports the fact of the high‐rate capability of Co‐SLMoS_2_/NOC. Notably, Co‐SLMoS_2_/NOC also shows huge advantages when comparing the obtained values of *D*_Li_^+^ with recently reported MoS_2_‐based anode materials (Table S7, Supporting Information).

Investigating the conversion reaction of Co‐SLMoS_2_/NOC in LIBs system is of great interest. As exhibited in the ex situ TEM observation (Figure 4d,e, and Figure S19, Supporting Information), after discharging to 0.01 V, well‐dispersed nanoparticles with an average size of ≈2 nm can be observed. The diffraction rings of (310) and (321) planes of Mo (JCPDS:42‐1120), (102) and (110) planes of Co (JCPDS: 05–0727), and (220), (622), and (640) planes of Li_2_S (JCPDS: 26–1188) displayed in the SAED pattern (Figure 4f) indicates that the nanoparticles produced in conversion reaction contain Mo and Co. Note that Co and Mo nanoparticles produced from Co‐SLMoS_2_/NOC can induce the formation of the space charge zone to store extra Li^+^.^[^ ^37^, ^52^ ^]^ Specifically, the d‐orbit electrons of Co and Mo are not full, spin‐polarized electrons can be injected into the d orbits of the metallic Co and Mo nanoparticles with a depth of the Thomas–Fermi screening length in an electric field. Meanwhile, lithium ions are stored at the surfaces of the Co and Mo nanoparticles to construct the space charge zone, thus leading to the spin‐polarized surface capacitance.^[^ ^37^ ^]^ An interesting feature of space‐charge storage is that it possesses the potential to be very fast, which is because ions and electrons in the space charge zone travel along separate pathways rather than in bulk storage where ions and electrons must travel in the same phase.^[^ ^52^ ^]^ To evidence the formation of the space charge zone, ex situ XPS spectra of Co‐SLMoS_2_/NOC electrode at various discharge/charge states are performed and exhibited in Figure 4g,h. In the pristine state, the binding energy of Mo 3d and Co 2p contains 229.0 eV (Mo 3d_5/2_), 232.0 eV (Mo 3d_3/2_), 778.7 eV (Co 2p_3/2_), and 793.7 eV (Co 2p_1/2_). When the electrode is fully discharged to 0.01 V, these characteristic peaks shift obviously to lower binding energy (228.3 eV, Mo^0^ 3d_5/2_; 231.3 eV, Mo^0^ 3d_3/2_; 777.8 eV, Co^0^ 2p_3/2_; 793.3 eV, Co^0^ 2p_1/2_), confirming the production of metallic Mo and Co due to the conversion reaction.^[^ ^37^, ^53^, ^54^ ^]^ In the fully charged state, these peaks show a further negative shift (227.9 eV, Mo^0^ 3d_5/2_; 230.8 eV, Mo^0^ 3d_3/2_; 777.0 eV, Co^0^ 2p_3/2_; and 792.0 eV, Co^0^ 2p_1/2_). This may be because numerous positively charged lithium ions stored on the surface of Mo and Co nanoparticles increase the binding energy. Therefore, the binding energy shows negative shift after the delithiation from the Mo and Co particle surface. These results demonstrate the formation of the space charge zone.^[^ ^37^ ^]^

Because the magnetism is sensitive to the change in the phase, structure, and electronic state of transition metal‐based materials,^[^ ^55^ ^]^ the Li‐ion storage behavior of the Co‐SLMoS_2_/NOC electrode is further studied by magnetic characterization. As shown in the ex situ magnetic hysteresis loops in Figure 4i, a pronounced increase in magnetization for Co‐SLMoS_2_/NOC can be detected from ≈0 emu g^−1^ (before cycling) to 12.6 emu g^−1^ (after discharging to 0.01 V), signaling the formation of Mo and Co nanoparticles due to the reduction of Mo^4+^ and Co^2+^.^[^ ^37^ ^]^ After charging to 3 V, the magnetization presents a further increase to 17.7 emu g^−1^. Specifically, many extra electrons stored in Mo and Co nanoparticles partially cancel out the spin majority bands of the 4d (Mo) and 3d (Co) energy levels, reducing the magnetization. Therefore, the magnetization shows a further increase after the delithiation from the surface of Mo and Co nanoparticles, which further confirms the formation of the space charge zone.^[^ ^37^ ^]^

To reveal the main responsible for the strong surface capacitance effect of the Co‐SLMoS_2_/NOC electrode, MLMoS_2_, FLMoS_2_/NOC, and Co‐FLMoS_2_/NOC electrodes are also investigated by ex situ TEM and XPS spectra. After the full discharge, the size of Mo nanoparticles generated from MLMoS_2_ is ≈10 nm (Figure S20, Supporting Information), while that from FLMoS_2_/NOC is ≈3.0 nm (Figure S21, Supporting Information), suggesting the nanoscale few‐layered structure of MoS_2_ and the presence of carbon matrix effectively inhibit the growth and aggregation of Mo nanoparticles during the conversion reaction. The smaller Mo nanoparticle with larger specific surface area can provide more interfaces for extra Li^+^ storage and result in a stronger surface‐capacitance effect,^[^ ^56^ ^]^ which is confirmed by XPS spectra and magnetic hysteresis loops (detailed discussion in Figures S22 and 23, Supporting Information). After introducing a small amount of Co doping, Co and Mo nanoparticles generated from Co‐FLMoS_2_/NOC show an average size of ≈3.0 nm (Figure S24, Supporting Information), which is similar to the size of Mo nanoparticles produced from FLMoS_2_/NOC (Figure S21, Supporting Information). However, the magnetization of Co‐FLMoS_2_/NOC is much higher than that of FLMoS_2_/NOC after discharge (0.56 emu g^−1^, FLMoS_2_/NOC; 6.6 emu g^−1^, Co‐FLMoS_2_/NOC) and charge process (1.31 emu g^−1^, FLMoS_2_/NOC; 11.1 emu g^−1^, Co‐FLMoS_2_/NOC), as shown in Figures S23 and S25 (Supporting Information). Theoretically, higher electrode magnetization enables more spin‐polarized electrons to inject into d orbits of transition metal,resulting in a larger space charge zone, thereby enhancing the spin‐polarized surface capacitance.^[^ ^37^ ^]^ This means that the strong spin‐polarized surface capacitance is mainly caused by the small amounts of Co nanoparticles. Notably, compared to Co‐FLMoS_2_/NOC electrode, Co‐SLMoS_2_/NOC electrode with a higher Co doping amount (Table S3, Supporting Information) produces more Co nanoparticles with a smaller average size of ≈2 nm after fully discharging (Figure 4e). The smaller nanoparticles generated from Co‐SLMoS_2_/NOC electrode are due largely to the smaller MoS_2_ microcell (lateral dimension×thickness: ≈6.5 × 0.4 nm, in Co‐SLMoS_2_/NOC, Figure 2l; ≈12×5 nm, in Co‐FLMoS_2_/NOC, Figure 2h) and the confinement of carbon matrix. With the synergistic effect of the smaller size and larger amount of Co nanoparticles, the Co‐SLMoS_2_/NOC electrode exhibits higher magnetization than the Co‐FLMoS_2_/NOC electrode after discharge (6.6 emu g^−1^, Co‐FLMoS_2_/NOC; 12.6 emu g^−1^, Co‐SLMoS_2_/NOC) and charge process (11.1 emu g^−1^, Co‐FLMoS_2_/NOC; 17.7 emu g^−1^, Co‐SLMoS_2_/NOC), thereby causing stronger spin‐polarized surface capacitance.

### 2.4 Practical Exploration of Co‐SLMoS2/NOC in LIBs

**Figure 5:**
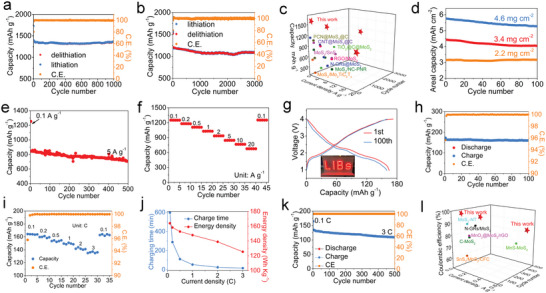
Cycling performances at a) 1 A g^−1^ and b) 5 A g^−1^ of Co‐SLMoS_2_/NOC, c) performance comparison of MoS_2_‐based anode materials, d) cycling performances of Co‐SLMoS_2_/NOC with different mass loadings at 0.1 A g^−1^. Cycling performance at 5 A g^−1^ (e) and rate performance (f) of Co‐SLMoS_2_/NOC with mass loadings of 4.6 mg cm^−2^. g–l) The electrochemical performances of full cells, g) charge/discharge curves at 0.1 C and the inserted optical image of light‐emitting diode array powered by a single full cell, h) cycling performances at 0.1 C, i) rate performance, j) relationship of charging time and energy density under different current densities, k) cycling performances at 3 C, l) performances comparison of full cells when using MoS_2_‐based materials as the anode.

We further explore the potential of Co‐SLMoS_2_/NOC for practical applications in view of its superior electrochemical performances and advanced Li‐ion storage mechanism. As can be seen, Co‐SLMoS_2_/NOC delivers a reversible capacity of 1372.2 mAh g^−1^ at 1 A g^−1^ over 1000 cycles with a high capacity retention of 98.9% (**Figure** **5**a). Even at 5 A g^−1^, Co‐SLMoS_2_/NOC still delivers a high capacity of 1084.3 mAh g^−1^ after 3000 charge–discharge cycles (90.1%, capacity retention, Figure 5b), denoting excellent stability during repeated rapid charge and discharge process. When comparing the performances of Co‐SLMoS_2_/NOC with recently reported MoS_2_‐based anode materials for LIBs (Figure 5c and Table S8, Supporting Information), Co‐SLMoS_2_/NOC shows huge advantages in terms of capacity, cyclability, and rate capability. In practice, the areal capacity is a crucial criterion to evaluate the application potential of the LIBs electrode materials. The areal capacity strongly depends on the areal mass loading of the electrode materials. Consequently, the cycling stability of the Co‐SLMoS_2_/NOC with active mass loading ranging from 2.2 to 4.6 mg cm^−2^ is tested (Figure 5d). Obviously, Co‐SLMoS_2_/NOC exhibits good stability with high mass loading, and especially a high areal capacity of 5.26 mAh cm^−2^ still remains for loading 4.6 mg cm^−2^ after 100 cycles at 0.1 A g^−1^ (Figure 5d). Even after long‐term cycling (500 cycles) at 5 A g^−1^, the electrode (4.6 mg cm^−2^) still maintains a capacity of 706.6 mAh g^−1^ with a capacity retention of 83.8% (Figure 5e). In addition, Figure 5f shows that the electrode with high loading (4.6 mg cm^−2^) also exhibits excellent rate capability (675.9 mAh g^−1^ at 20 A g^−1^), further confirming the potential value of Co‐SLMoS_2_/NOC in LIBs application. Afterward, the full cells are assembled with commercial LiFePO_4_ cathode to further assess the application potential of the Co‐SLMoS_2_/NOC anode, which successfully powers the light‐emitting diode array (Figure 5g). During the first and 100th cycles, the full cell presents almost overlapped charge/discharge curves, which indicates stable electrode structure upon cycling (Figure 5g). A high reversible capacity of 160.0 mAh g^−1^ can be obtained with a capacity retention of 97.2% after 100 cycles at 0.1 C (Figure 5h). As being tested at 1 C, the capacity can still remain at 141.1 mAh g^−1^ after 200 cycles (Figure S26, Supporting Information), signaling the outstanding cycle stability of the full cell. The full cell also exhibits excellent rate performance. Upon gradually elevating the current density, the galvanostatic discharge–charge voltage profiles of the full cell remain stable (Figure S27, Supporting Information), and a high capacity of 135.8 mAh g^−1^ is obtained at 3 C (Figure 5i). After reducing the current density back to 0.1 C, the capacity of the full cell rapidly increases to 163.0 mAh g^−1^, implying a fine capacity recovery. After charging for 15.3 min at 3 C, the full cell shows a high energy density of 125.5 Wh kg^−1^ (the calculation of the energy density presented in Supporting information), which is 76.5% of the energy density at 0.1 C (164.0 Wh kg^−1^; Figure 5j). Moreover, a high reversible capacity of 109.6 mAh g^−1^ can still be maintained after 500 cycles at 3 C (81.9%, capacity retention, Figure 5k). The full‐cell performances are superior to previous reports on full cells when MoS_2_‐based materials as the anode (Figure 5l and Table S9, Supporting Information), fully demonstrating the excellent high‐rate stability and high practical potential of Co‐SLMoS_2_/NOC.

**Figure 6:**
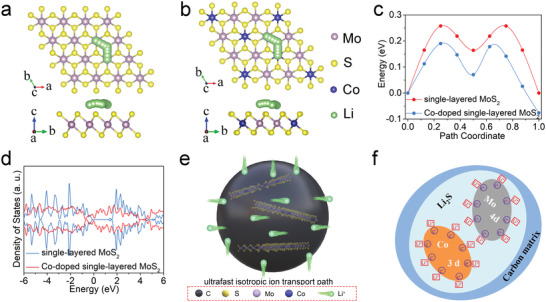
The migration paths of lithium ions in a) pure single‐layered MoS_2_ and b) Co‐doped single‐layered MoS_2_, c) the diffusion energy barrier of lithium ions in pure and Co‐doped single‐layered MoS_2_. d) DOS of pure and Co‐doped single‐layered MoS_2_. Schematic diagram of e) Li‐ion diffusion in Co‐SLMoS_2_/NOC and f) the spin‐polarized surface capacitance effect.

We further investigate the behavior of Li‐ion diffusion on MoS_2_ plane of Co‐SLMoS_2_/NOC. The DFT calculations are carried out to provide Li‐ion diffusion energy barriers for undoped and Co‐doped single‐layered MoS_2_, in which the doping amount of Co obeys the XPS results (Table S2, Supporting Information). **Figure** **6**a‐c shows the relative energy curves along the diffusion pathways, as can be seen, the Li‐ion diffusion barrier of Co‐doped single‐layered MoS_2_ at each site is much lower than that of pure single‐layered MoS_2_, implying Co doping can hugely improve the Li‐ion diffusion kinetics, which results in an excellent Li‐ion transport capability. In addition, the doped Co atoms can obviously reduce the bandgap of single‐layered MoS_2_ to enable high carrier transportation, as presented in the calculated density of states (DOS) in Figure 6d. The pure single‐layered MoS_2_ possesses a semiconductor feature (≈1.40 eV, bandgap), while the metallic property is achieved after introducing Co atoms in the MoS_2_ lattices (0 eV, bandgap). Co‐doping can therefore improve carrier transportation in single‐layered MoS_2_. This is mainly due to Co doping can interfere with the surrounding atoms and induce lattice distortion of MoS_2_, thus tuning the electronic structure.^[^ ^57^, ^58^ ^]^ Figure 6e shows the Li‐ion diffusion mechanism in Co‐SLMoS_2_/NOC. The amorphous carbon possesses a large number of defects, through which the ions are transported to the MoS_2_ layers and stored at every active spot on the surface of both sides. The presence of the Co atoms in the MoS_2_ layers accelerates this process as the Co atoms present electronegativity due to the presence of the excess electrons. Meanwhile, Co doping generates a strong spin‐polarized surface capacitance effect on Co nanoparticles (Figure 6f). The Li storage performance is effectively enhanced by the above factors.

### 2.5 Electrochemical Performances for SIBs and PIBs

**Figure 7:**
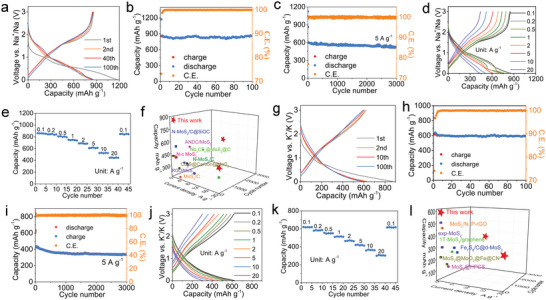
Electrochemical performances of Co‐SLMoS_2_/NOC electrode for SIBs (a–f) and PIBs (g–l). a) Charge/discharge curves for different cycles. Cycling performances at 0.1 A g^−1^ (b) and 5 A g^−1^ (c), respectively. d) Charge/discharge curves at different current densities. e) Rate performances. f) Performance comparison of MoS_2_‐based SIBs anode materials. g) Charge/discharge curves for different cycles. Cycling performances at 0.1 A g^−1^ (h) and 5 A g^−1^ (i), respectively. j) Charge/discharge profiles at different current densities. k) Rate performances. l) Performance comparison of MoS_2_‐based PIBs anode materials.

The electrochemical performances of Co‐SLMoS_2_/NOC as anode material for SIBs (**Figure** **7**a‐f) and PIBs (Figure 7g‐l) are also evaluated. As can be seen, Co‐SLMoS_2_/NOC delivers high initial discharge/charge capacities with relatively high initial CE (1186.9/866.2 mAh g^−1^, 73.0%, SIBs; 883.0/620.9 mAh g^−1^, 70.3%, PIBs), outstanding cycle stability (100.3% (SIBs) and 96.0% (PIBs) capacity retention after 100 cycles at 0.1 A g^−1^; 86.0% (SIBs) and 79.1% (PIBs) capacity retention after 3000 cycles at 5 A g^−1^), and excellent rate capability (445.3 mAh g^−1^ at 20 A g^−1^, SIBs; 303.2 mAh g^−1^ at 20 A g^−1^, PIBs). When compared with previously reported MoS_2_‐based anode materials for SIBs and PIBs, Co‐SLMoS_2_/NOC displays the best comprehensive electrochemical performances in terms of capacity, cyclability, and rate performances (Figure 7f, Table S10, Supporting Information, SIBs; Figure 7l, Table S11, Supporting Information, PIBs). Consequently, the above results fully demonstrate the huge advantages of Co‐SLMoS_2_/NOC in rechargeable ion batteries, especially for LIBs, SIBs, and PIBs.

## 3 Conclusions

In summary, a novel charge‐driven interlayer expansion strategy is proposed for fabricating single‐layered MoS_2_ based on Co doping, which induces strong electrostatic repulsion to break the confinement of interlayer van der Waals forces, and is demonstrated by DFT calculation. Co‐doped single‐layered MoS_2_ with fully exposed MoS_2_ basal planes, increased intrinsic EC, low lithium ion diffusion energy barrier, and vanishing interlayer ion diffusion barrier hugely boosts ion transport and storage capability. The Co‐doped single‐layered MoS_2_ uniformly dispersed into N, O co‐doped carbon matrix is highly stable during cycling, which shows an ultralow volume expansion of 10.3% after 100 cycles. The doped magnetic Co atom in single‐layered MoS_2_ lattices can transform into ultrasmall Co nanoparticles (≈2 nm) during conversion reaction, which induces strong spin‐polarized surface capacitance to further enhance ion transport and storage. As a consequence, the Co‐SLMoS_2_/NOC electrode delivers superior electrochemical performances than all the previous reports on MoS_2_‐based anode for Li/Na/K‐ion batteries. The proposed single‐layered MoS_2_ synthesis strategy may be generalized to other 2D layered materials stabilized by weak van der Waals attraction. This work may provide new insight into the design of single‐layered transition metal dichalcogenides for rechargeable ion batteries.

## Conflict of Interest

The authors declare no conflict of interest.

## Supporting information

Supporting Information

## Data Availability

The data that support the findings of this study are available in the supplementary material of this article.
