# Supplementary material for: Single‐Layered MoS2 Fabricated by Charge‐Driven Interlayer Expansion for Superior Lithium/Sodium/Potassium‐Ion‐Battery Anodes
Source: Adv Sci (Weinh). 2023 Mar 22;10(15):2207234. doi: 10.1002/advs.202207234 (PMC10214217; doi:10.1002/advs.202207234)
Supplement: Supplementary file 1 — Supporting Information [file ADVS-10-2207234-s001.pdf]

ADVANCED SCIENCE
Open Access

# Supporting Information

for *Adv. Sci.*, DOI 10.1002/advs.202207234

Single-Layered $MoS_2$ Fabricated by Charge-Driven Interlayer Expansion for Superior Lithium/Sodium/Potassium-Ion-Battery Anodes

*Zhenwei Li, Meisheng Han*, Yuanbo Zhang, Fu Yuan, Ying Fu* and Jie Yu**

ADVANCED SCIENCE
Open Access

# Supporting Information

for *Adv. Sci.*, DOI 10.1002/advs.202207234

Single-Layered $MoS_2$ Fabricated by Charge-Driven Interlayer Expansion for Superior Lithium/Sodium/Potassium-Ion-Battery Anodes

*Zhenwei Li, Meisheng Han*, Yuanbo Zhang, Fu Yuan, Ying Fu* and Jie Yu**

Supporting Information

**Single-layered $MoS_2$ Fabricated by Charge-Driven Interlayer Expansion for Superior Lithium/Sodium/Potassium-Ion-Battery Anodes**

Zhenwei Li[1a,b], Meisheng Han[1a,c]*, Yuanbo Zhang[b], Fu Yuan[a], Ying Fu[a]*, and Jie Yu[a,b]*

[a]Songshan Lake Materials Laboratory, Dongguan, Guangdong 523808, China

[b]Guangdong Provincial Key Laboratory of Semiconductor Optoelectronic Materials and Intelligent Photonic Systems, Shenzhen Engineering Lab for Supercapacitor Materials, School of Material Science and Engineering, Harbin Institute of Technology, Shenzhen, University Town, Shenzhen 518055, China

[c]Department of Mechanical and Energy Engineering, Southern University of Science and Technology, Shenzhen 518055, China

*Corresponding Author.

E-mail address: jyu@hit.edu.cn (J. Yu); hanms@sustech.edu.cn; fuying@sslab.org.cn

[1]Both authors contributed equally to this work.

## Experimental Section

### *Synthesis of MLMoS$_2$, FLMoS$_2$/NOC, Co-FLMoS$_2$/NOC, and Co-SLMoS$_2$/NOC*

For the preparation of samples, the distinct amount of cobalt naphthenate/$(NH_4)_2MoS_4$/DMF (0/1.5/0, $MoS_2$; 0/0.5/1.0 g, FLMoS$_2$/NOC; 0.3/0.50/0.70 g, Co-FLMoS$_2$/NOC; 0.5/0.50/0.50 g, Co-SLMoS$_2$/NOC) were added in the self-made vessels. For exploring the effect of excessive Co doping on sample structure, the distinct amount of cobalt naphthenate/$(NH_4)_2MoS_4$/DMF of 0.6/0.50/0.40 g was also added in the vessel. After that, these vessels were sealed in an Ar-filled glove box, subsequently transferred to a tube furnace, kept at 500 °C for 10 min in an Ar flow, and then cooled to ambient temperature naturally to obtain these samples. All these above-mentioned chemical reagents were bought from Macklin.

### *Characterizations*

SEM (Hitachi S-4700), TEM (FEI Talos F200x), EELS (FEI, Titan Themis G2), XPS (Thermo Scientific Escalab Xi$^+$), TGA (Pyris I, PerkinElmer), and EA (Perkinelmrr 2400 Series II), as well as XRD (D/max-2500/PC, Rigaku) and Raman spectroscopy (Horiba LabRam HR Evolution) were applied to analyze the microstructure and chemical composition of samples. Four-probe tester (Probes Tech RTS-8, China) was used to measure the electrical conductivity of samples. The BET (ASAP 2020, HD88) was applied to measure the surface area and pore size distribution of these samples. The magnetic hysteresis curves were measured by the physical property measurement system (PPMS, Quantum Design).

### *EC measurements*

First, the obtained powders were added in a hollow cylinder mould with two electrodes on each ends, which were connected with a digital multimeter (Keithley 2001, USA) and followed by compressing powders into slices. During compressing the electrical resistance was observed constantly. When the electrical resistance kept stable the mould was opened, obtaining slices. Subsequently, the electrical conductivity of slices was measured using a four-probe tester (Probes Tech RTS-8, China).

***Electrochemical measurements***

The working electrodes were prepared by mixing 80 wt% of active materials, 10 wt% of acetylene black, and 10 wt% of polyvinylidene fluoride (PVDF) into N-methyl pyrrolidone under mechanical stirring to to make the slurries. The as-prepared slurries were uniformly coated on the copper foil, and dried at 120°C under vacuum for 10 h. 2032 coin-type cells contain working electrode, counter/reference electrode (lithium foil), and separator, as well as electrolyte, which were assembled in an argon-filled glove box. Celgard 2400 membrane was used as separator absorbing electrolyte (1 M $LiPF_6$ in a mixture of ethylene carbonate (EC)/diethylene carbonate (DEC)/dimethyl carbonate (DMC) at a volume ratio of 1:1:1 with 5 wt% fluoroethylene carbonate (FEC)). For SIBs, using Whatman glass fiber as separator absorbing electrolyte (1 M $NaPF_6$ in a mixture of EC and DEC 1:1 (vol%) with 5 wt% FEC). For PIBs, using Whatman glass fiber as separator absorbing electrolyte (1.0 M potassium bis(fluorosulfonyl)imide dissolved in the mixture of EC and DEC with a volume ratio of 1:1 with 5 wt% FEC). The mass loading density of active materials in each

electrode slice is 1.2 mg $cm^{-2}$. Land CT2001A battery-test system (Wuhan Land Electronic co., China) was applied to evaluate the electrochemical performances of cells at the charge/discharge current density of 0.1-20 A $g^{-1}$ between 0.01 and 3 V. A CHI 760D electrochemical workstation (Shanghai CH Instruments Co., China) was utilized to measure CV and electrochemical impedance spectroscopy (EIS). CV measurements were performed at scanning rate of 0.1-1 mV $s^{-1}$ from 0.01 to 3 V (vs. $Li/Li^{+}$). EIS was carried out from $10^5$ to $10^{-2}$ Hz with an amplitude of 5 mV. Coin-type full cells compose of $LiFePO_4$ (cathode materials) and prepared nanocomposites (anode). Before assembling, the anode was activated for the three cycles at 0.1 C in half cells to enhance its first CE. The cathode was fabricated by coating a mixture of 95.0 wt% $LiFePO_4$, 2.5 wt% PVDF, and 2.5 wt% acetylene black on Al foil. The full cell has an N/P ratio of ~1.06. The mass loading of active material for anodes is 1.8 mg $cm^{-2}$, and that of cathodes is 15.3 mg $cm^{-2}$. The cycling and rate tests of the full cell were carried out at 0.1-3 C (1 C = 170 mA $g^{-1}$) between 1.0-4.0 V. All cells were tested at room temperature.

***Computational method***

All the DFT calculations were conducted based on the Vienna Ab-inito Simulation Package (VASP).[1, 2] The electron-ion interactions were described by the Projected Augmented-Wave (PAW) potentials, while the exchange-correlation interactions were calculated by employing the Perdew-Burke-Enzerhof (PBE) pseudopotentials of Generalized Gradient Approximation (GGA).[3,4] DFT-D3 method was employed to calculate the van der Waals (vdW) interaction.[5] The plane-wave energy cutoff was

set as 450 eV. The convergence threshold was set as $1.0\times10^{-5}$ eV in energy and 0.02 eV per Angstrom in force. The Brillouin zone was sampled with 4 × 4 × 1 k-points. A vacuum space of 30 Å was inserted in z direction to avoid interactions between periodic images. The migration energy barrier of lithium ions were performed by employing climbing image nudged elastic band (CI-NEB) method, and the force was converged to 0.03 eV/Å.

***Calculation of the gravimetric energy density***

The gravimetric energy density of the full cell can be calculated from the follow equation.

$$\text{Gravimetric energy density (Wh kg}^{-1}\text{)} = \left( \frac{Cc \times V}{\left(m_{active} + m_{inactive}\right)} \right)$$

Where $V$-nominal voltage (2.2 V); $C_c$-cell capacity (2.4 mAh); $m_{active}$-active mass (16.6 mg) of cathode and anode; $m_{inactive}$-inactive mass (15.6 mg) of the conductive agent, electrolyte, and binder, as well as separator and current collectors.

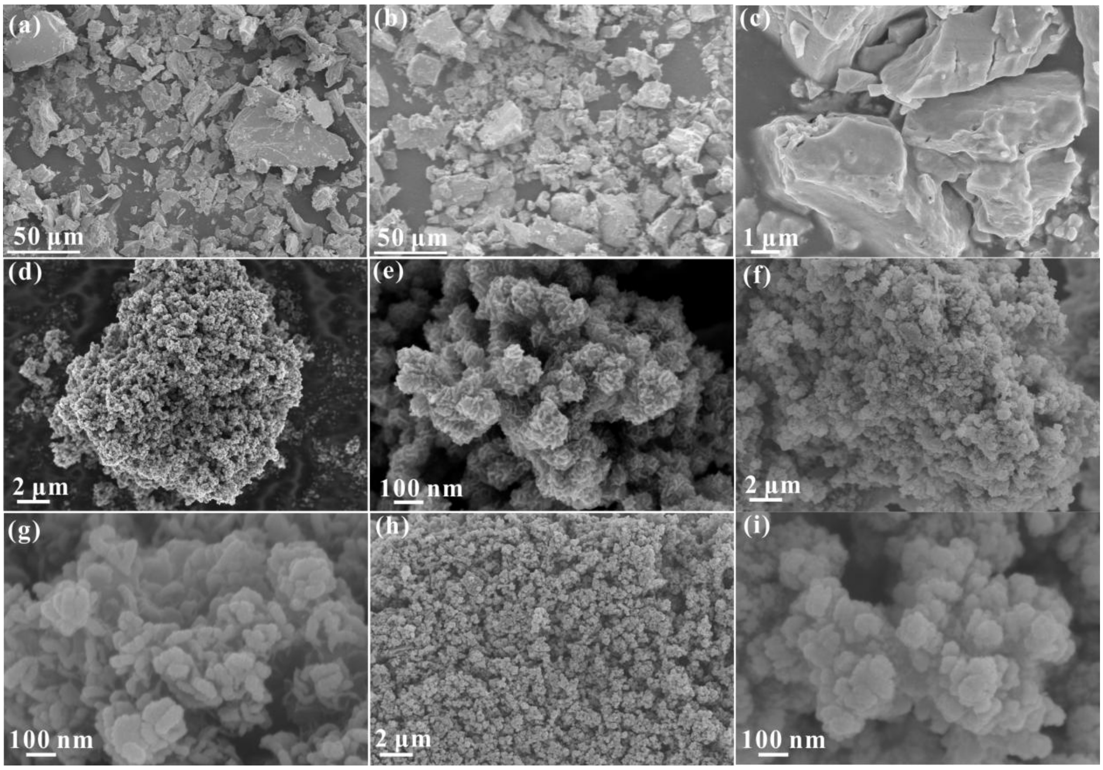


**Figure S1.** SEM images of $(NH_4)_2MoS_4$ (a), $MLMoS_2$ (b, c), $FLMoS_2$/NOC (d, e), Co-$FLMoS_2$/NOC (f, g), and Co-$SLMoS_2$/NOC (h, i).

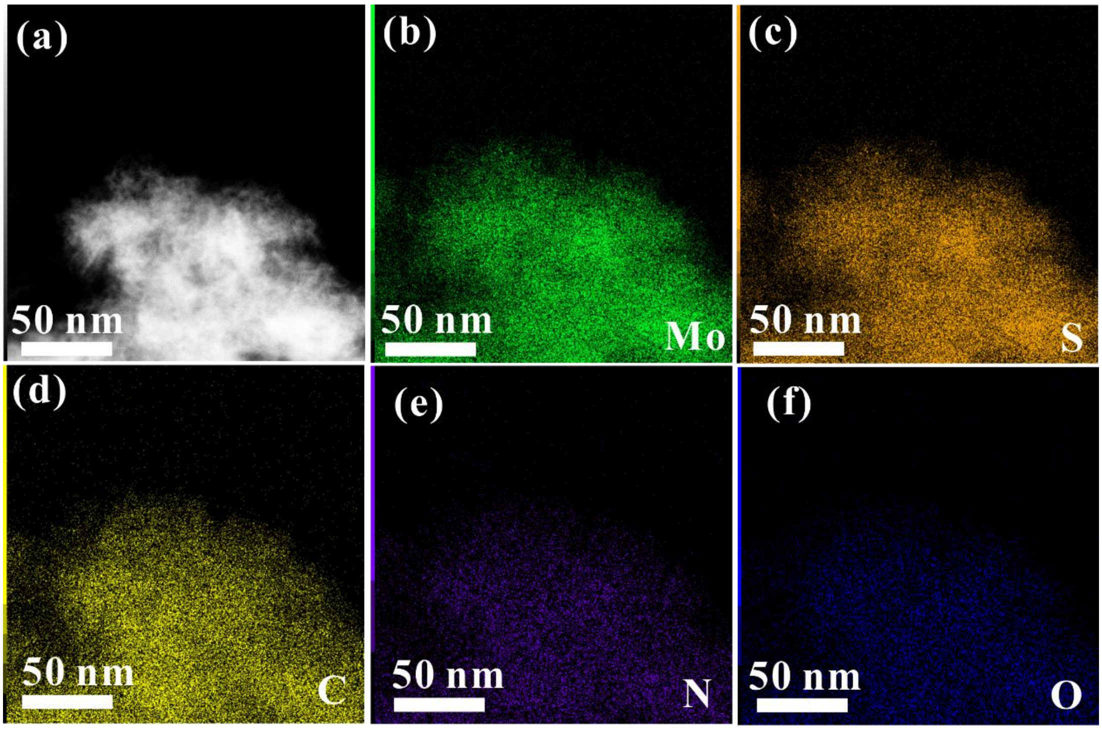


**Figure S2.** (a) HAADF image and corresponding EDS mapping images of Mo (b), S (c), C (d), N (e), and O (f) of FLMo$S_2$/NOC.

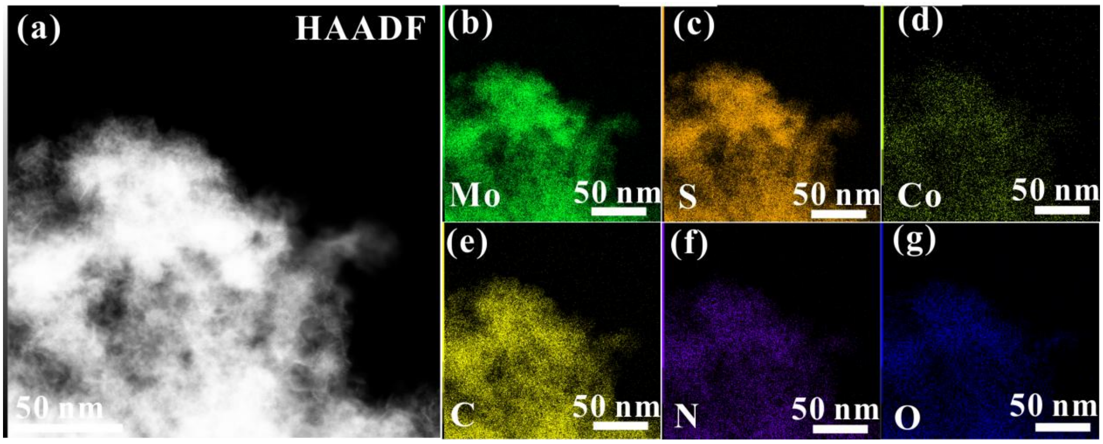


**Figure S3.** (a) HAADF image and corresponding EDS mapping images of Mo (b), S (c), Co (d), C (e), N (f), and O (g) of Co-FLMo$S_2$/NOC.

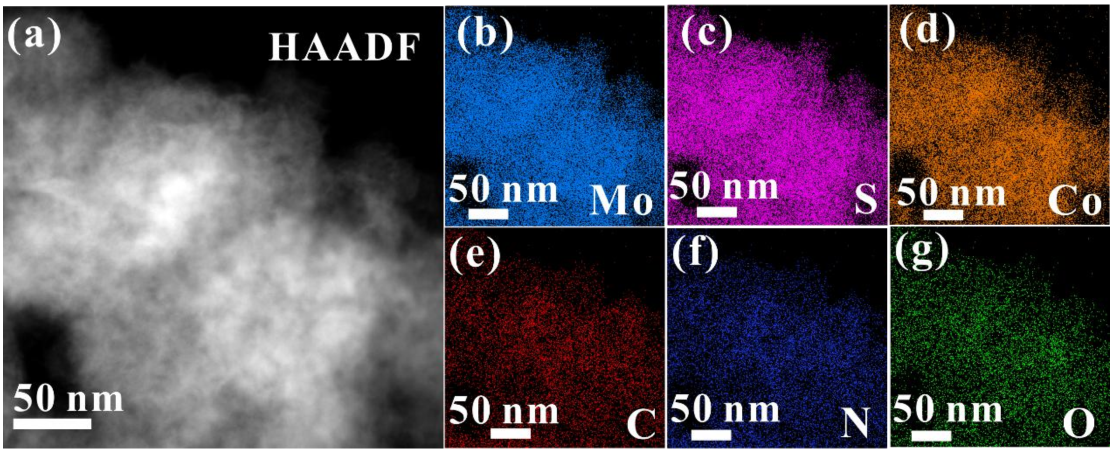


**Figure S4.** (a) HAADF image and corresponding EDS mapping images of Mo (b), S (c), Co (d), C (e), N (f), and O (g) of Co-SLMo$S_2$/NOC.

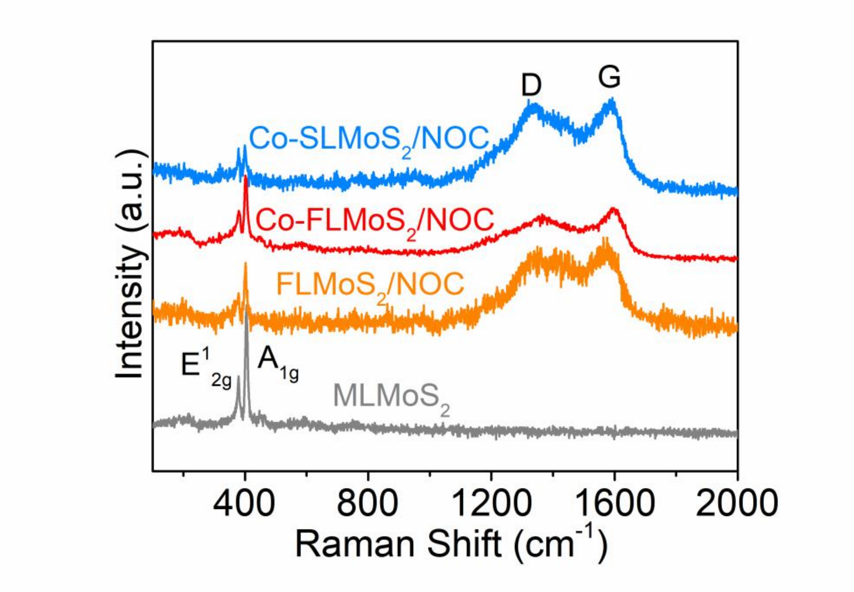


**Figure S5.** Raman spectra of the obtained samples.

**Table S1** The intensity ratios ($I_D/I_G$) of $FLMoS_2$/NOC, Co-$FLMoS_2$/NOC, and Co-$SLMoS_2$/NOC.

| Samples | $FLMoS_2$/NOC | Co-$FLMoS_2$/NOC | Co-$SLMoS_2$/NOC |
|---|---|---|---|
| $I_D/I_G$ | 0.94 | 0.91 | 0.93 |

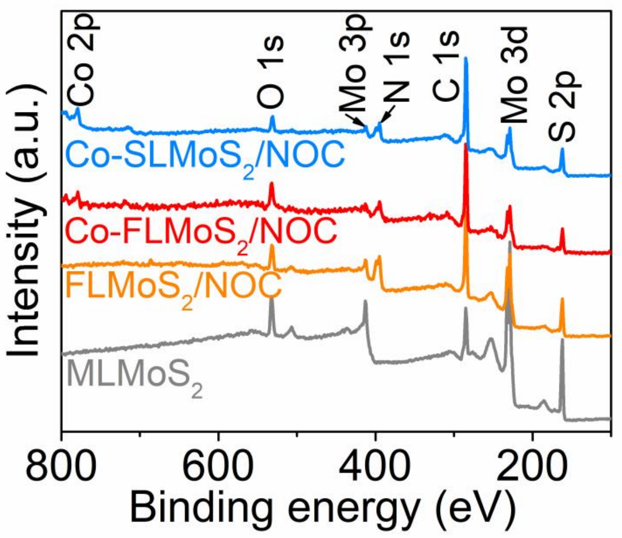


**Figure S6.** XPS survey peaks of the obtained samples.

**Table S2** Fitting results of XPS spectra of all the samples.

| Samples | Mo (at%) | S (at%) | Co (at%) | C (at%) | N (at%) | O (at%) |
|---|---|---|---|---|---|---|
| $MLMoS_2$ | 30.55 | 61.44 | 0 | 4.26 | 0 | 3.75 |
| $FLMoS_2$/NOC | 5.13 | 10.39 | 0 | 70.13 | 9.48 | 4.87 |
| Co-$FLMoS_2$/NOC | 3.82 | 9.45 | 0.76 | 74.40 | 7.56 | 4.01 |
| Co-$SLMoS_2$/NOC | 2.67 | 8.45 | 1.42 | 78.60 | 5.62 | 3.96 |

The doping amount of N and O elements in the carbon materials should be calculated by N (at%)/(C (at%)+N (at%)+O (at%))*100% and O (at%)/(C (at%)+N (at%)+O (at%))*100%, respectively. The doping amount of Co elements in the $MoS_2$ materials should be calculated by Co (at%)/Co (at%)+Mo (at%)+S (at%))*100%, respectively. The specific doping amount is represented in Tables S3 and S4.

**Table S3** The doping amount of Co elements in the $MoS_2$ materials

| Samples | Co (at%) |
|---|---|
| Co-FLMo$S_2$/NOC | 5.42 |
| Co-SLMo$S_2$/NOC | 11.30 |

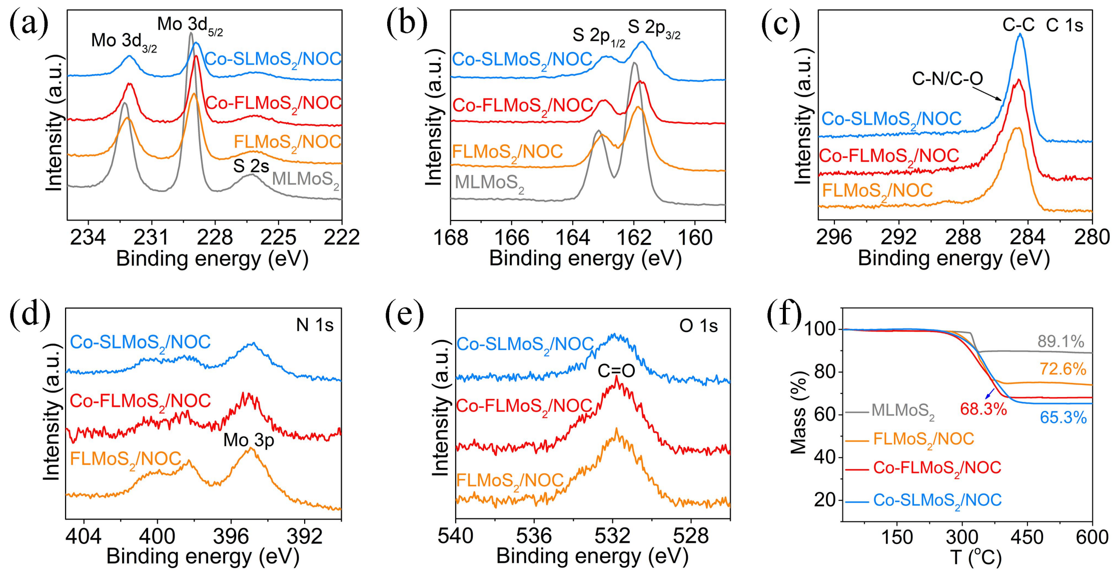


**Figure S7.** (a-e) high-resolution XPS spectra of (a) Mo 3d, (b) S 2p, (c) C 1s, (d) N 1s, and (e) O 1s, and (f) TGA curves of the prepared samples.

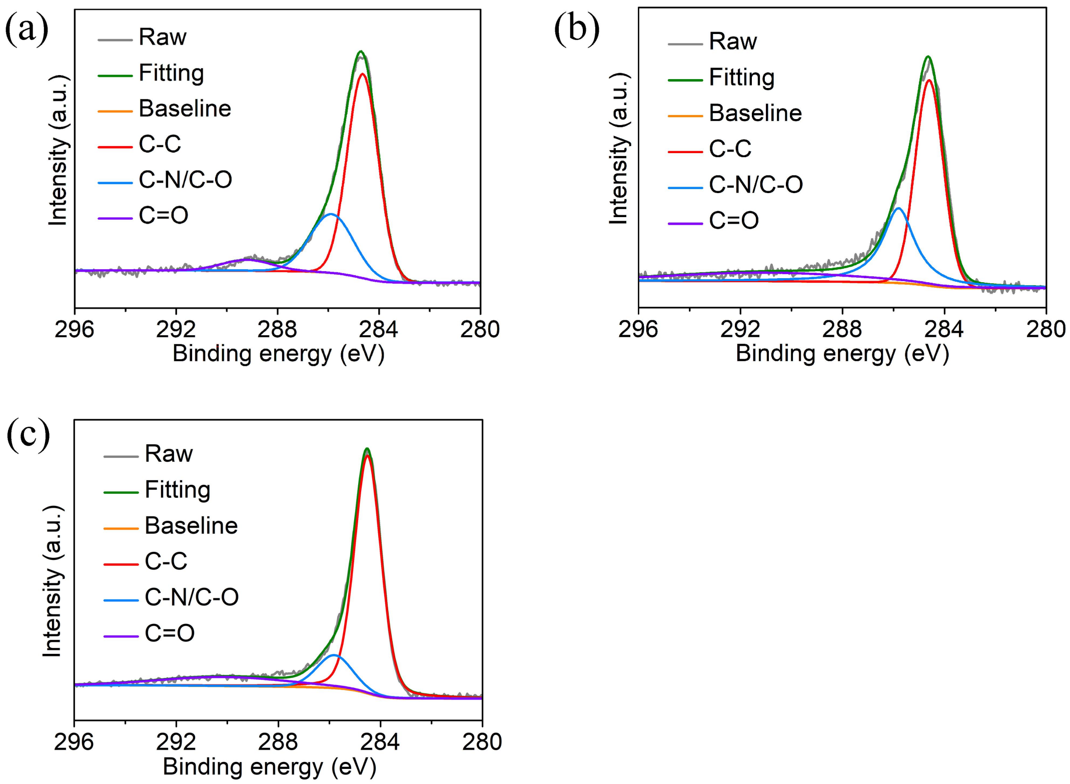


**Figure S8.** The fitted spectra of C 1s. (a) FLMoS$_2$/NOC, (b) Co-FLMoS$_2$/NOC, (c) Co-SLMoS$_2$/NOC.

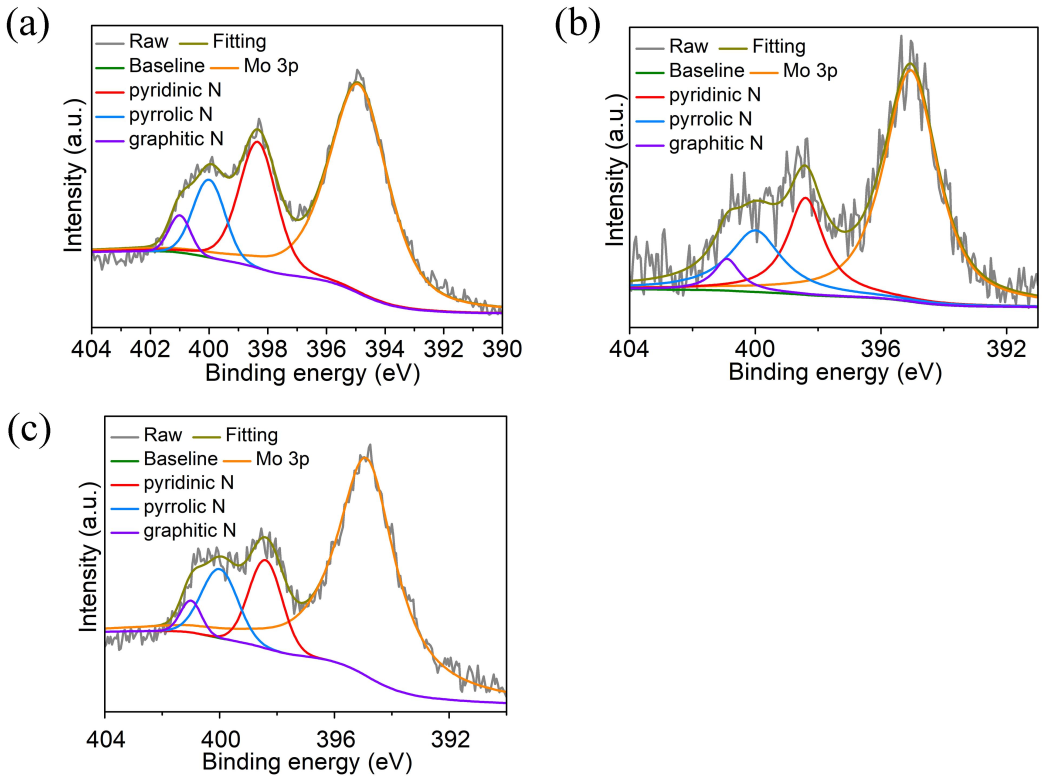


**Figure S9.** The fitted spectra of N 1s. (a) FLMoS$_2$/NOC, (b) Co-FLMoS$_2$/NOC, (c) Co-SLMoS$_2$/NOC.

**Table S4** The doping amount of N and O elements in the carbon materials

| Samples | N (at%) | O (at%) |
|---|---|---|
| FLMoS$_2$/NOC | 11.22 | 5.76 |
| Co-FLMoS$_2$/NOC | 8.79 | 4.66 |
| Co-SLMoS$_2$/NOC | 6.43 | 4.53 |

**Table S5** The EC of the obtained samples.

| Samples | Electrical conductivity (S $cm^{-1}$) |
|---|---|
| **$MLMoS_2$** | $1.3 \times 10^{-3}$ |
| **$FLMoS_2$/NOC** | 4.8 |
| **Co-$FLMoS_2$/NOC** | 22.4 |
| **Co-$SLMoS_2$/NOC** | 58.1 |

The higher electrical conductivity is more favorable for charge transport.

**Table S6** The elemental analysis results of the obtained samples.

| Samples | Co (wt%) | C (wt%) | N (wt%) | O (wt%) | Mo (wt%) | S (wt%) |
|---|---|---|---|---|---|---|
| $MLMoS_2$ | 0 | ~0 | 0 | ~0 | 59.6 | 40.4 |
| $FLMoS_2$/NOC | 0 | 14.3 | 2.2 | 1.4 | 49.5 | 32.7 |
| Co-$FLMoS_2$/NOC | 5.1 | 16.2 | 1.9 | 1.2 | 41.4 | 34.2 |
| Co-$SLMoS_2$/NOC | 12.1 | 18.1 | 1.5 | 1.2 | 32.3 | 34.8 |

The C, N, O, and S contents in the composites were measured using O/N/H and C/S elemental analyzers. The Co contents in the composites were tested by inductively coupled plasma mass spectrometer. The content of Mo was calculated as a difference to 100 wt%. It can be seen that the mass percentages of N, O co-doped carbon matrix are 17.9, 19.3, and 20.8wt%, corresponding to $FLMoS_2$/NOC, Co-$FLMoS_2$/NOC, and Co-$SLMoS_2$/NOC, respectively.

After heating in air atmosphere, the increase of the mass is ascribed to the oxidation of Mo and Co into $MoO_3$ and $Co_2O_3$, while the decrease of the mass is attributed to the oxidation of C, N into $CO_2$ and $NO_2$ and the mass loss of O. Consequently, the final residual products are $MoO_3$ and/or $Co_2O_3$.

According to the element analysis results (Table S6), the mass of the final residual products for $MLMoS_2$, $FLMoS_2$/NOC, Co-$FLMoS_2$/NOC, and Co-$SLMoS_2$/NOC should be 89.4, 74.3, 69.2, and 65.4wt%, respectively.

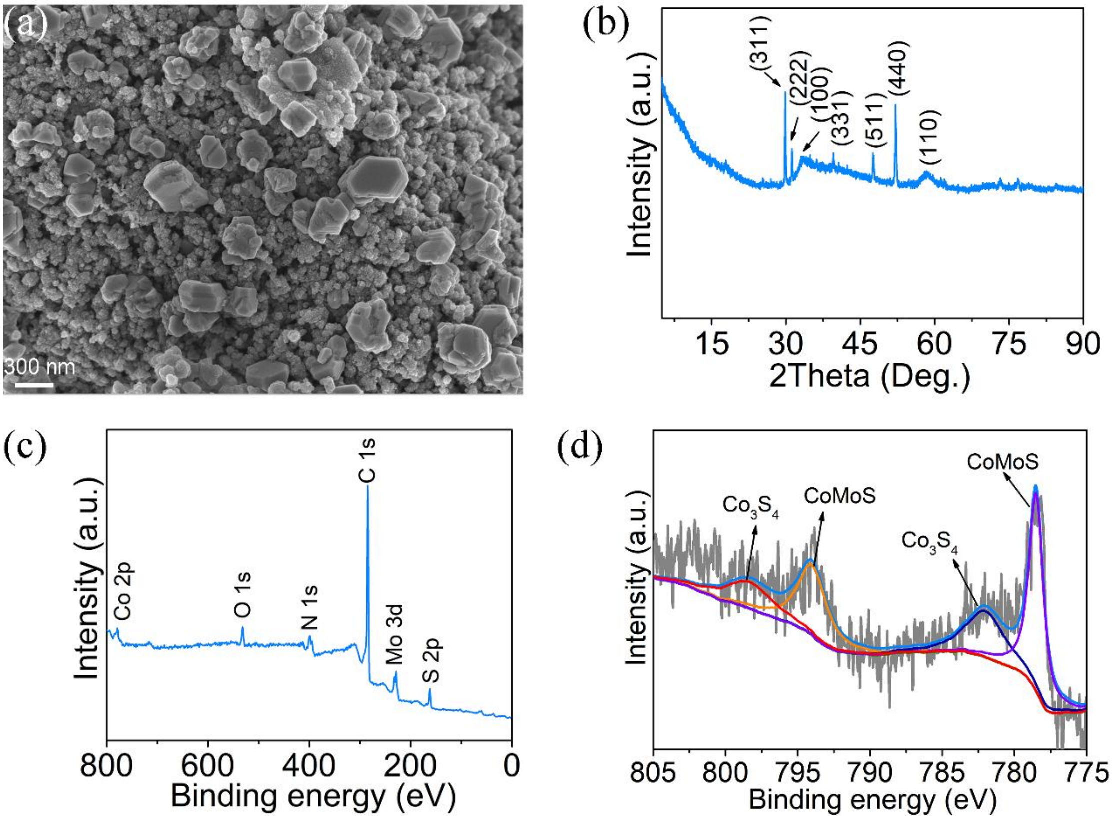


**Figure S10.** (a) SEM images, (b) XRD patterns, (c) XPS survey peaks, and (d) high-resolution XPS spectra of Co of the prepared sample with excessive Co doping.

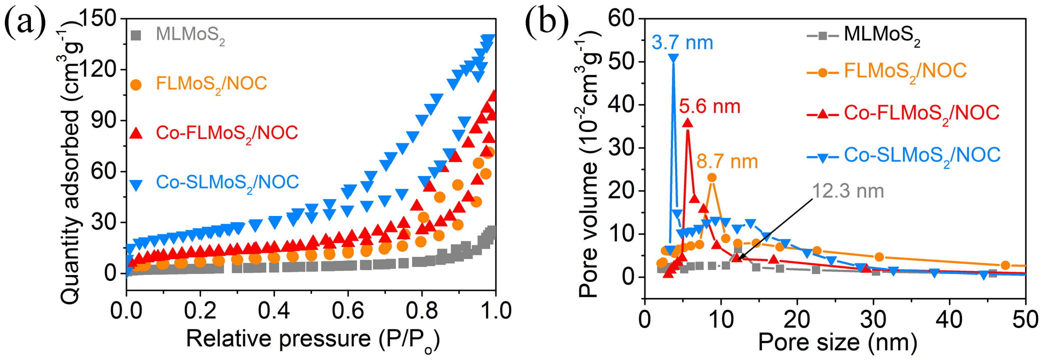


**Figure S11.** (a) Nitrogen adsorption/desorption isotherms and (b) pore size distribution of the obtained samples.

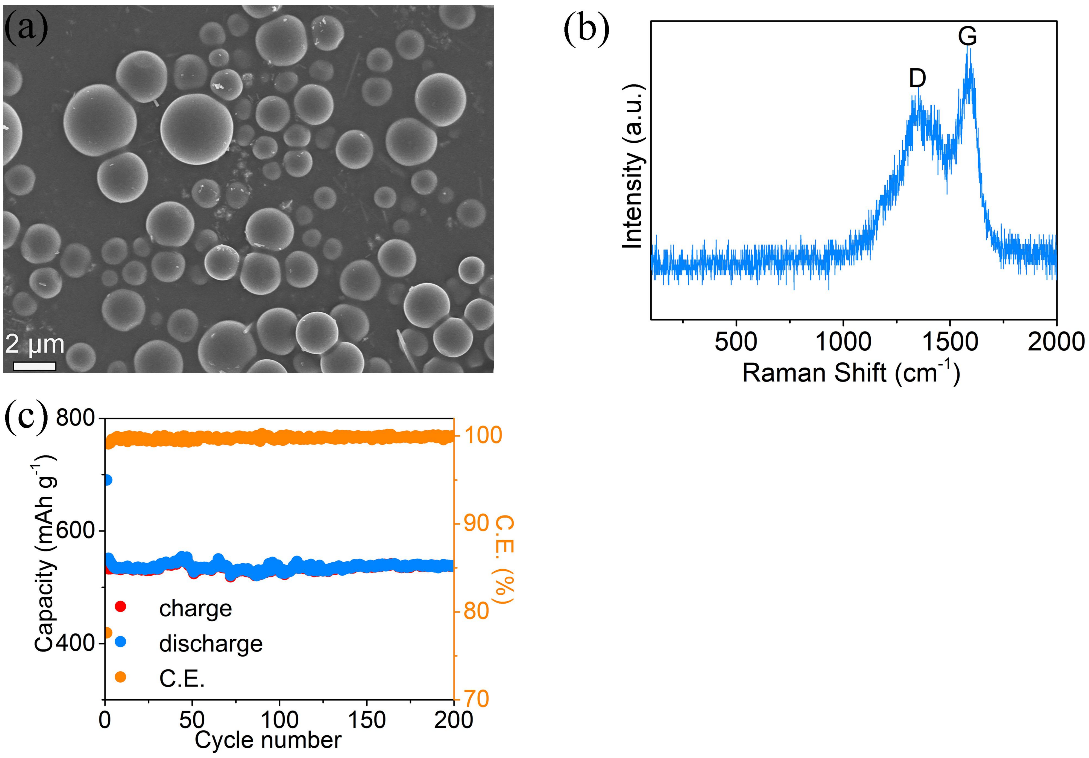


**Figure S12.** (a) SEM image, (b) Raman spectrum, and (c) cycling performance at 0.1 A $g^{-1}$ of the sample obtained by pyrolysis of DMF in the sealed vessel at 500 °C for 0.5 h.

To obtain the capacity of the N, O co-doped carbon matrix, DMF is thermally decomposed in the sealed vessel at 500 °C for 0.5 h to form N, O co-doped carbon matrix. As can be seen in Figure S12a, the pyrolysis products show a micro-sized spherical structure. Raman spectra deliver two obvious characteristic peaks of the D-band (~1339 $cm^{-1}$) and G-band (~1588 $cm^{-1}$) of carbon materials (Figure S12b). When used as anode material for LIBs (Figure S12c), N, O co-doped carbon shows a first charging capacity of 535.9 mAh $g^{-1}$. After 200 cycles, a capacity can still remain with a high capacity retention of 100.5 %, demonstrating good cycle stability.

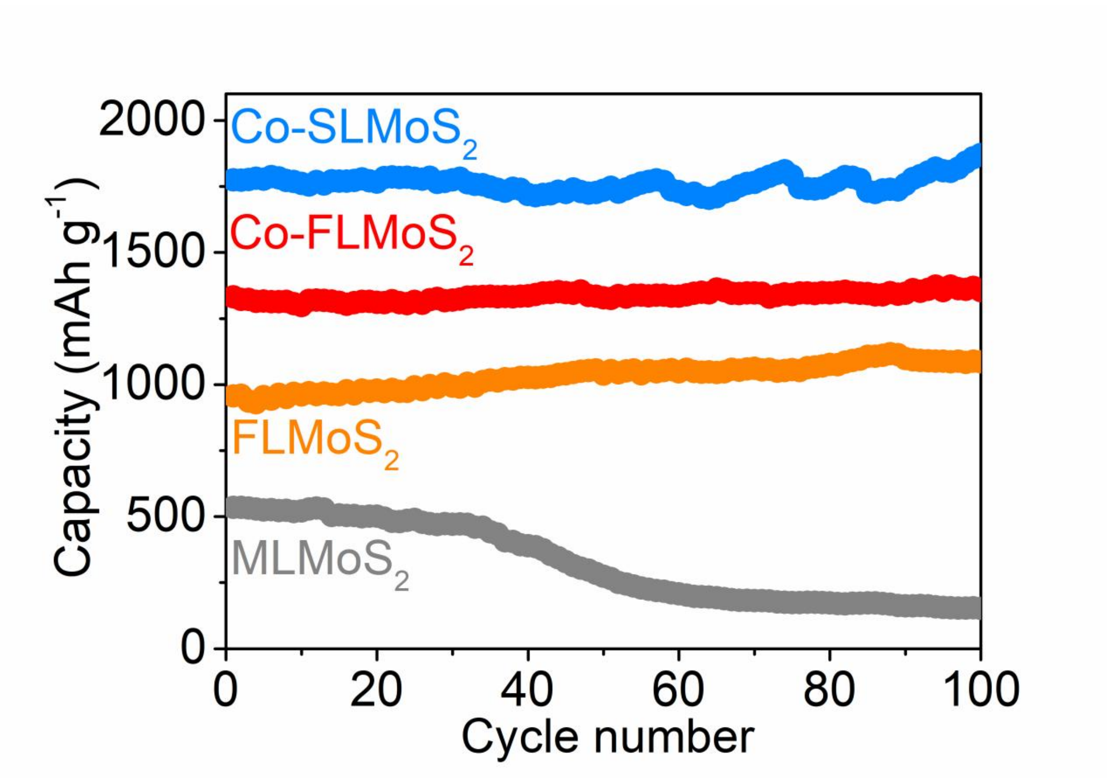


**Figure S13.** The cycling performances of the obtained samples at 0.1 A $g^{-1}$.

According to the results in Figure S12, we obtain the capacity of N, O co-doped carbon matrix during cycling. Therefore, the capacity of $MLMoS_2$, $FLMoS_2$, Co-$FLMoS_2$, and Co-$SLMoS_2$ in corresponding samples can be calculated approximately. The capacity is calculated based on [the total capacity (Figure 3b, in the manuscript) minus the capacity of the N, O co-doped carbon matrix multiplies mass percentages of N, O co-doped carbon materials] divides the mass percentage of $FLMoS_2$, Co-$FLMoS_2$, and Co-$SLMoS_2$. As can be seen in Figure S13, the first charging capacity of $MLMoS_2$, $FLMoS_2$, Co-$FLMoS_2$, and Co-$SLMoS_2$ can be calculated as 534.6, 956.4, 1332.1, and 1775.0 mAh $g^{-1}$, respectively.

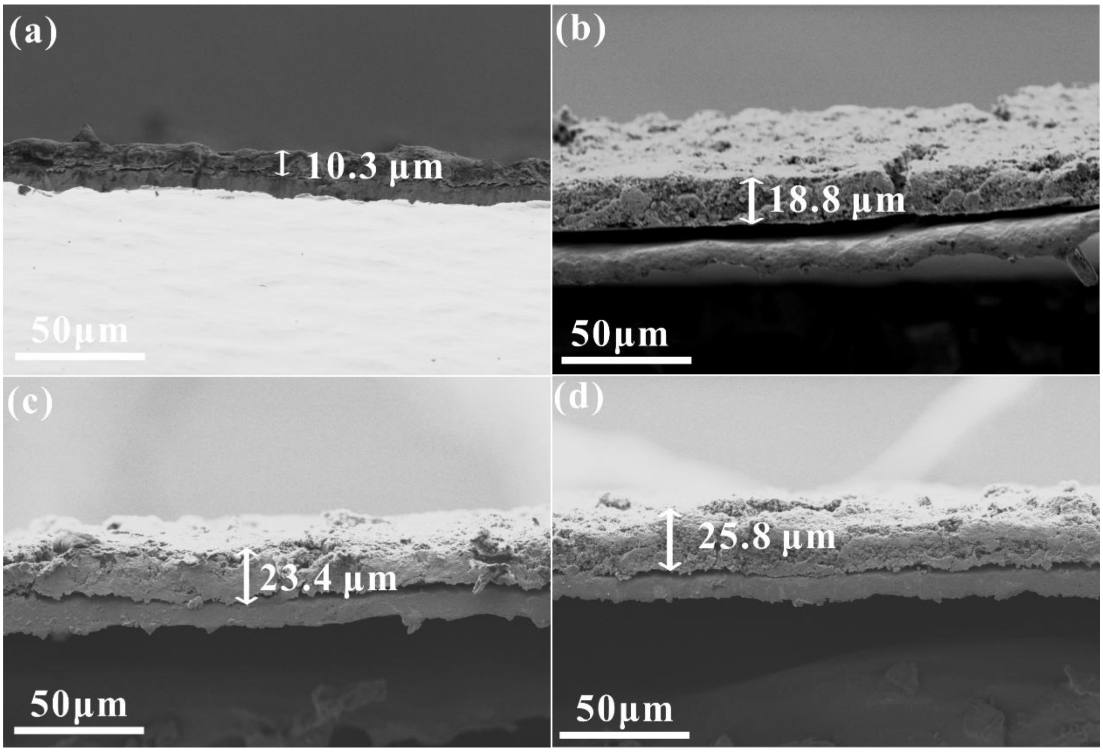


**Figure S14.** Cross-sectional SEM images of $MLMoS_2$ (a, b) and Co-$SLMoS_2$/NOC (c, d) electrodes. (a, c) before cycles and (b, d) after 100 cycles.

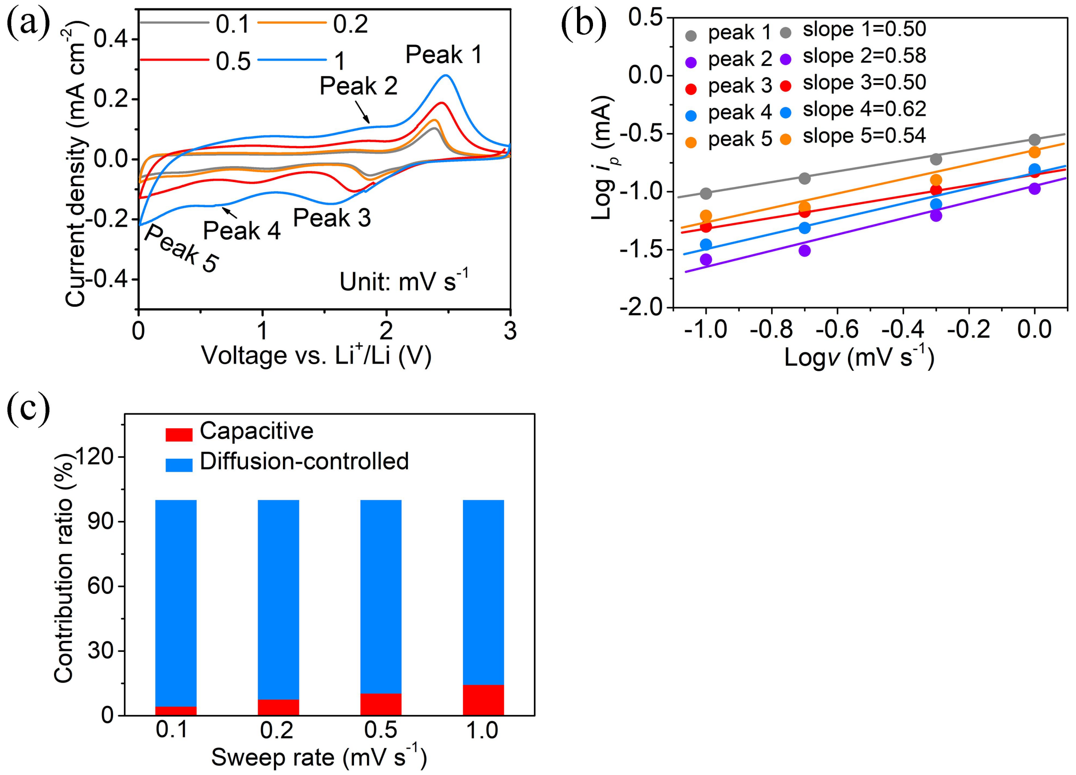


**Figure S15.** (a) CV curves of $MLMoS_2$ for LIBs at different sweep rates; (b) Log $i_p$ against Log $v$ at peaks 1-5; (c) the percentages of pseudocapacitive contribution at different sweep rates.

To comprehend the electrochemical kinetics and quantitative analysis of $MLMoS_2$ in detail, CV profiles at various scan rates (0.1-1.0 mV $s^{-1}$) are measured. Apparently, the CV profiles exhibit similar shapes during the reduction and oxidation processes with the increase of the scan rate (Figure S15a). The peak current ($i$) and the scan rate ($v$) obey the following equation.[6]

$$i = av^b \tag{1}$$

where a and b are empirical constants. Especially, the b-value of 0.5 belongs to a complete diffusion-controlled behavior and the b-value of 1.0 indicates an absolute capacitive-controlled process. The b-values of the marked peaks 1-5 in both cathodic and anodic processes for $MLMoS_2$ are 0.50, 0.58, 0.50, 0.62, and 0.54, respectively, as presented in Figure S15b. This indicates that the lithium-ion storage dynamics of the $MLMoS_2$ electrode include diffusion-controlled and pseudocapacitive-controlled

mechanisms. Moreover, the two processes contributions are quantified by the known equation.[7]

$$i(V) = k_1 v + k_2 v^{1/2} \quad (2)$$

where i(V), $k_1v$, and $k_2v^{1/2}$ stand for the total current at a fixed potential, the pseudocapacitance behavior, and diffusion-controlled process, respectively. The percentage of capacitive contribution only shows an slightly increasing trend from 4.3 to 14.1% with the increase of scan rate from 0.1 to 1 mV $s^{-1}$ (Figure S15c), indicating diffusion-controlled process dominants the capacity.

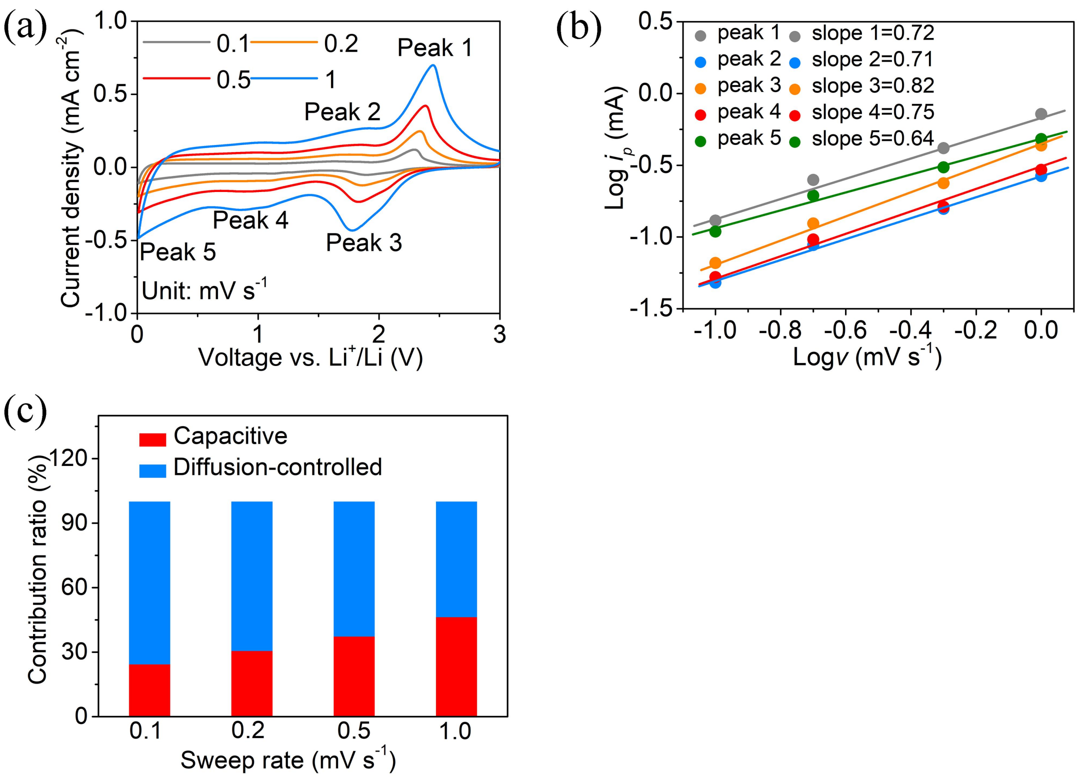


**Figure S16.** (a) CV curves of FLMoS$_2$/NOC for LIBs at different sweep rates; (b) Log $i_p$ against Log $v$ at peaks 1-5; (c) the percentages of pseudocapacitive contribution at different sweep rates.

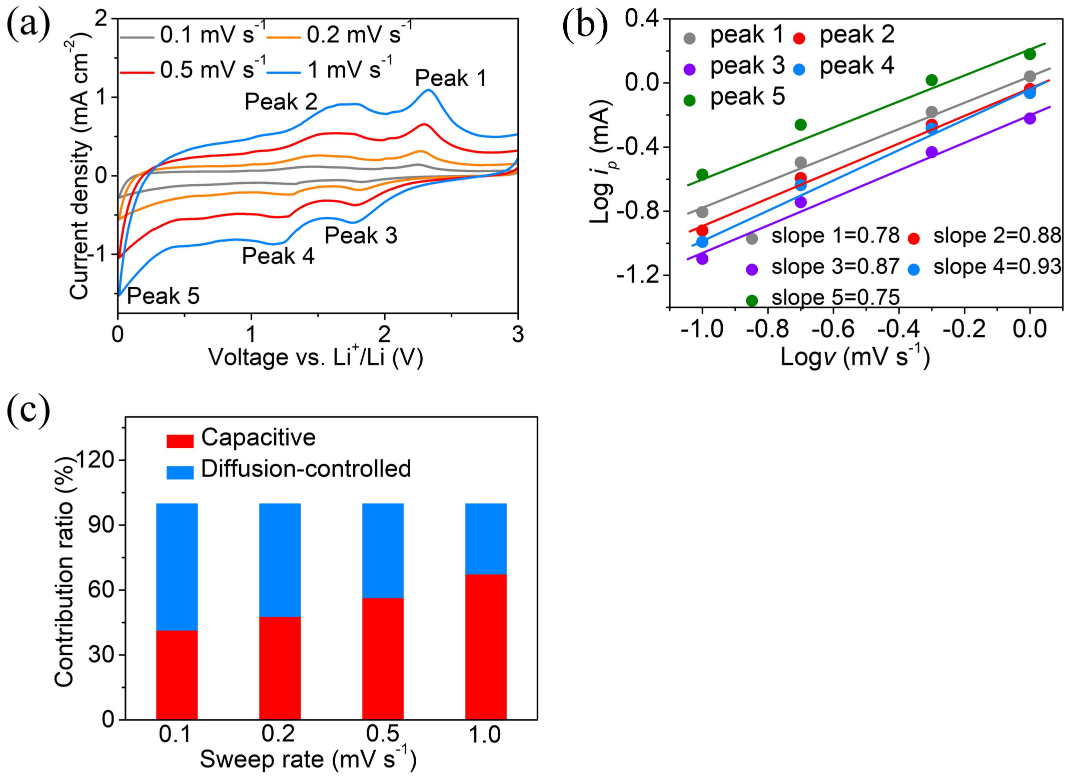


**Figure S17.** (a) CV curves of Co-FLMo$S_2$/NOC for LIBs at different sweep rates; (b) Log $i_p$ against Log $v$ at peaks 1-5; (c) the percentages of pseudocapacitive contribution at different sweep rates.

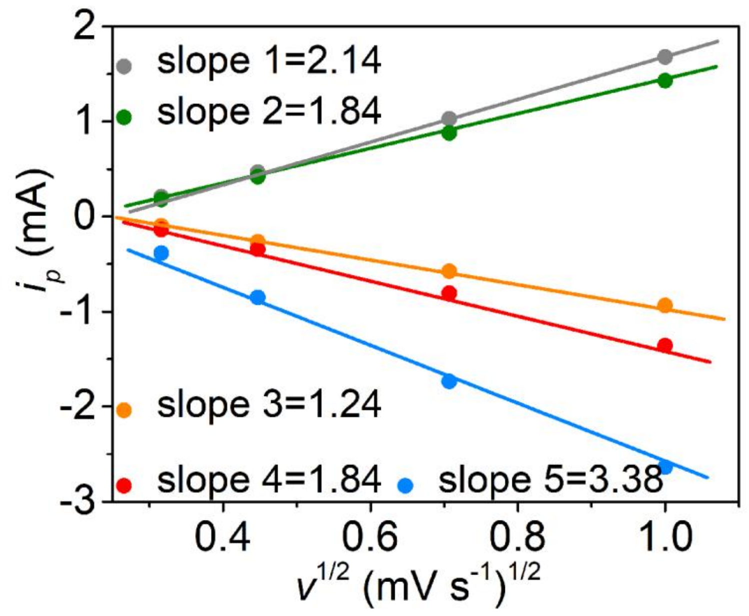


**Figure S18.** $i_p$ versus $v^{1/2}$ at peaks 1-5 of Co-SLMo$S_2$/NOC.

**Li-Ion Concentration Calculation**

At first, the unit cell volume of $MoS_2$ is calculated to be "a (3.16 Å) × b (3.16 Å) × c (12.29 Å) × sin120° = 106.28 × $10^{-24}$ $cm^3$", thus 1 $cm^3$ includes 1/106.28 × $10^{-24}$ = 9.41 × $10^{21}$ unit cells. The discharge capacity of Co-SLMo$S_2$/NOC can reach about 1520.1 mAh $g^{-1}$ during the initial several cycles, which corresponds to ~ 2.3 $Li^+$ insertion into $MoS_2$ per formula unit to form the $Li_{2.3}MoS_2$. In addition, every $MoS_2$ unit cell includes two molecules, and each molecule includes 2.3 Li-ions, 1 $cm^3$ contains 9.41 × $10^{21}$/6.02 × $10^{23}$ × 2 × 2.3 = 7.19 × $10^{-2}$ mol Li-ions. As a result, the Li-ion concentration in the Co-SLMo$S_2$/NOC electrode is about 7.1 × $10^{-2}$ mol $cm^{-3}$.

**Table S7.** Comparison of $D_{Li+}$(cm$^2$ s$^{-1}$) of the samples prepared in this work with the recently reported $MoS_2$-based LIB anode materials. The comparison value is the average value in Figure 5e.

| Samples | $D_{Li+}$ | Average $D_{Li+}$ | References |
|---|---|---|---|
| Co-SL$MoS_2$/NOC | $2.65\times10^{-10}$-$1.97\times10^{-9}$ | $1.12\times 10^{-9}$ | **This work** |
| MnS-$MoS_2$ | $10^{-14}$-$10^{-13}$ | 5x$10^{-14}$ | Adv. Funct. Mater. **2021**, 31, 2007132 |
| $MoS_2$ | $10^{-15}$-$10^{-14}$ | 5x$10^{-15}$ | Adv. Funct. Mater. **2021**, 31, 2007132 |
| $MoS_2$/C | 4.48x $10^{-18}$ | 4.48x$10^{-18}$ | Chem. Eng. J. **2019**, 372, 665–672 |
| Mn-doped $MoS_2$/C | 2.51x$10^{-16}$ | 2.51x$10^{-16}$ | Chem. Eng. J. **2019**, 372, 665–672 |
| $MoS_2$-C | 1x$10^{-15}$ -1x$10^{-9}$ | 5x$10^{-13}$ | ACS Appl. Mater. Interfaces **2016**, 8, 22168−22174 |
| $TiO_2$/$MoS_2$ | 3.12x$10^{-14}$-6.67x$10^{-14}$ | 4.9x$10^{-14}$ | J. Alloys Compounds **2021**, 892, 162075 |

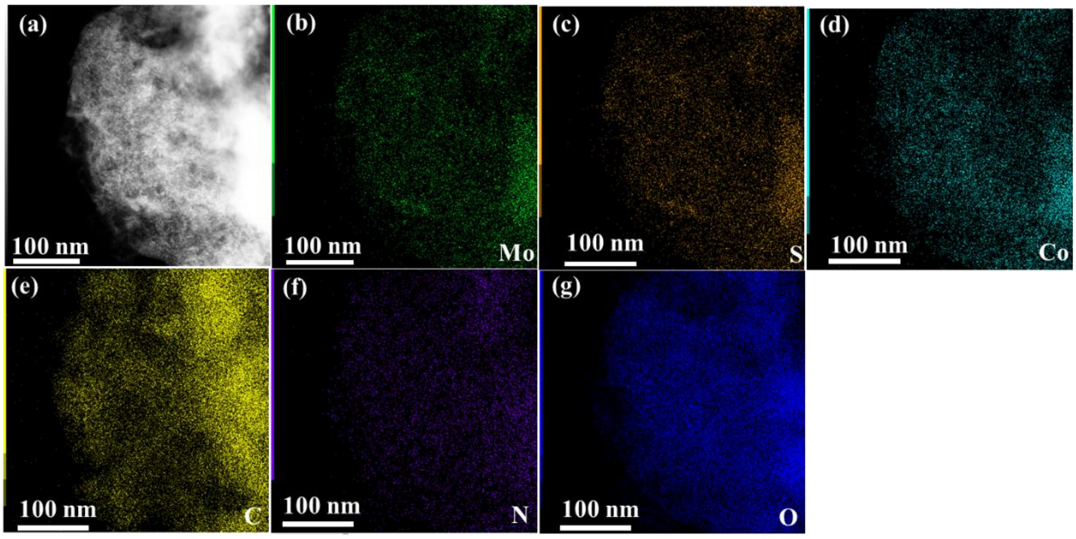


**Figure S19.** TEM characterizations of Co-SLMo$S_2$/NOC electrode after complete lithiation: (a) HAADF, and its corresponding EDS mapping images of Mo (b), S (c), Co (d), C (e), N (f), and O (g).

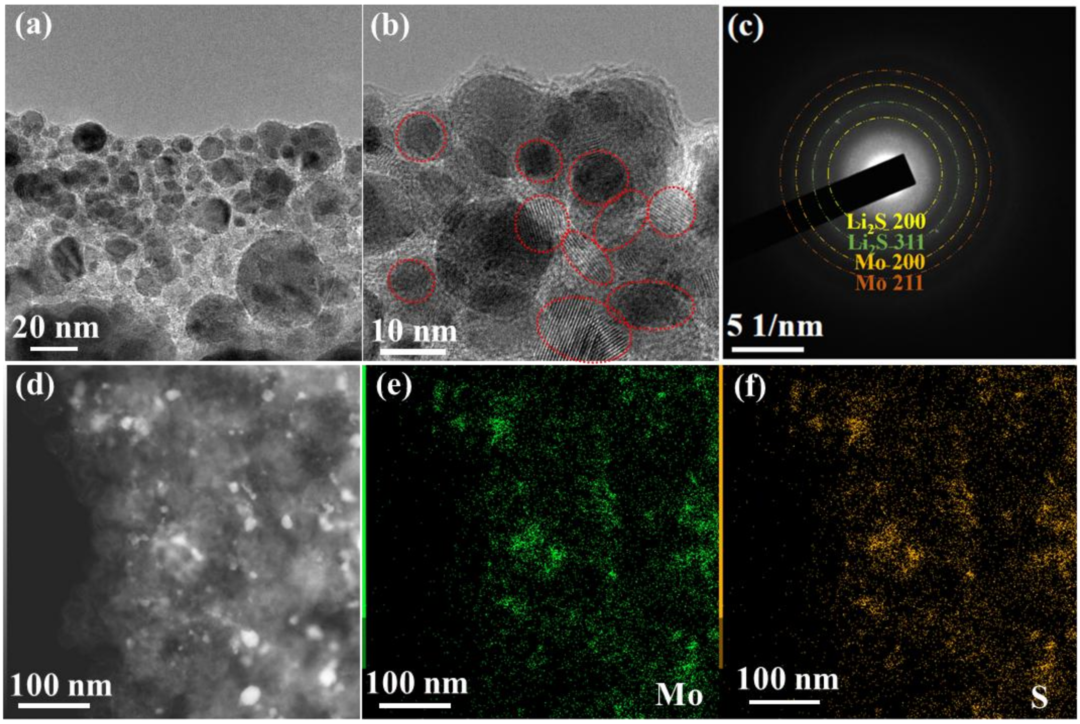


**Figure S20.** *Ex-situ* TEM characterizations of $MLMoS_2$ electrodes after discharging to 0.01 V. (a,b) TEM images, (c) corresponding SAED, (d) HAADF and corresponding EDS mapping images of Mo (e) and S (f).

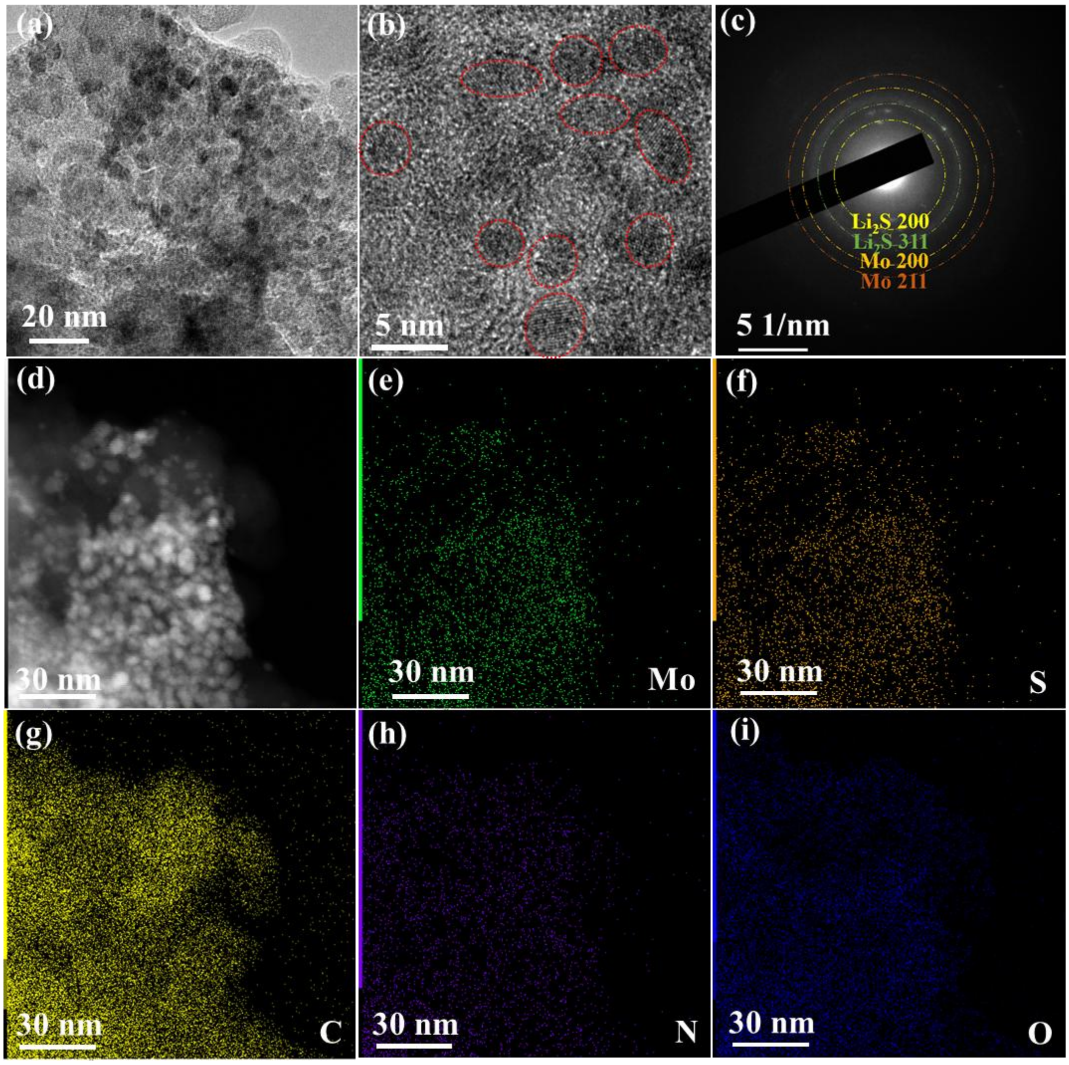


**Figure S21.** *Ex-situ* TEM characterizations of FLMoS$_2$/NOC electrodes after discharging to 0.01 V. (a,b) TEM images, (c) corresponding SAED, (d) HAADF and corresponding EDS mapping images of Mo (e), S (f), C (g), N (h), and O (i).

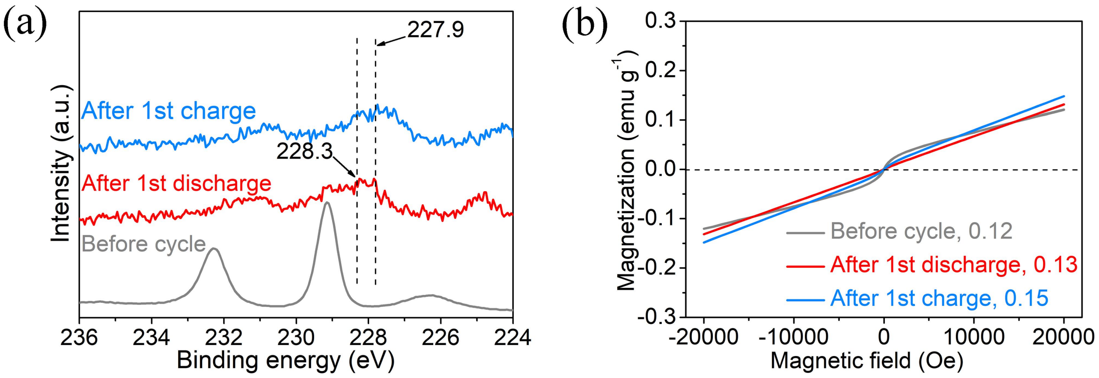


**Figure S22.** *Ex-situ* (a) Mo 3d XPS and (b) magnetic hysteresis loops of $MLMoS_2$ electrodes after discharging to 0.01 V and charging to 3 V.

As can be seen in Figure S22a, the characteristic peaks of Mo 3d shift remarkably to lower binding energy (228.3 eV, $Mo^0$) after discharging to 0.01 V, further suggesting the formation of metal Mo. Besides, a further negative shift of these peaks is detected (227.9 eV, $Mo^0$) when charging back to 3 V, which arises from the delithiation from the Mo surface. The pristine electrode shows a magnetization of 0.12 emu $g^{-1}$ (Figure S22b), while an increased magnetization of 0.13 emu $g^{-1}$ is obtained after discharging to 0.01 V, which is addressed to the formation of superparamagnetic Mo nanoparticles. The magnetization increases to 0.15 emu $g^{-1}$ after charging to 3 V because of the delithiation from the Mo surface.

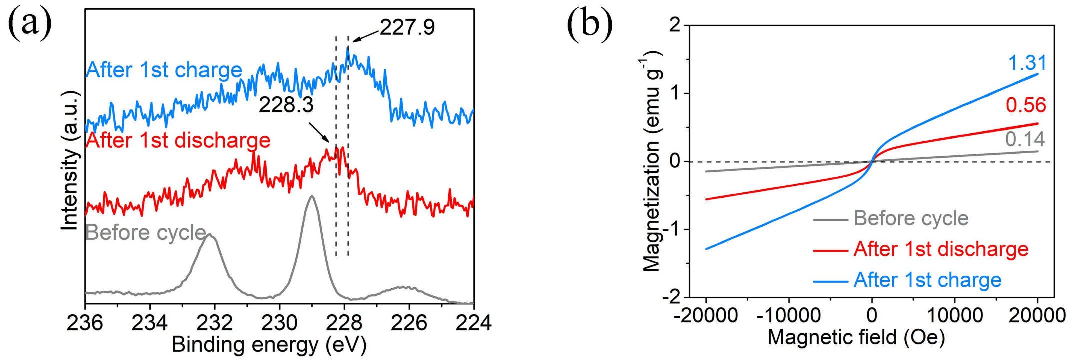


**Figure S23.** *Ex-situ* (a) Mo 3d XPS and (b) magnetic hysteresis loops of FLMo$S_2$/NOC electrodes after discharging to 0.01 V and charging to 3 V.

As can be seen in Figure S23a, the characteristic peaks of Mo 3d shift remarkably to lower binding energy (228.3 eV, $Mo^0$) after discharging to 0.01 V, further suggesting the formation of metal Mo. Besides, a further negative shift of these peaks is detected (227.9 eV, $Mo^0$), which arises from the delithiation from the Mo surface. The pristine electrode shows a magnetization of 0.14 emu $g^{-1}$ (Figure S23b), while an increased magnetization of 0.56 emu $g^{-1}$ is obtained after discharging to 0.01 V, which is addressed to the formation of superparamagnetic Mo nanoparticles. Clearly, the magnetization increases to 1.31 emu $g^{-1}$ after charging to 3 V because of the delithiation from the Mo surface. Consequently, the above results evidence the Li ions are stored on the Mo surface to construct the space charge zone.

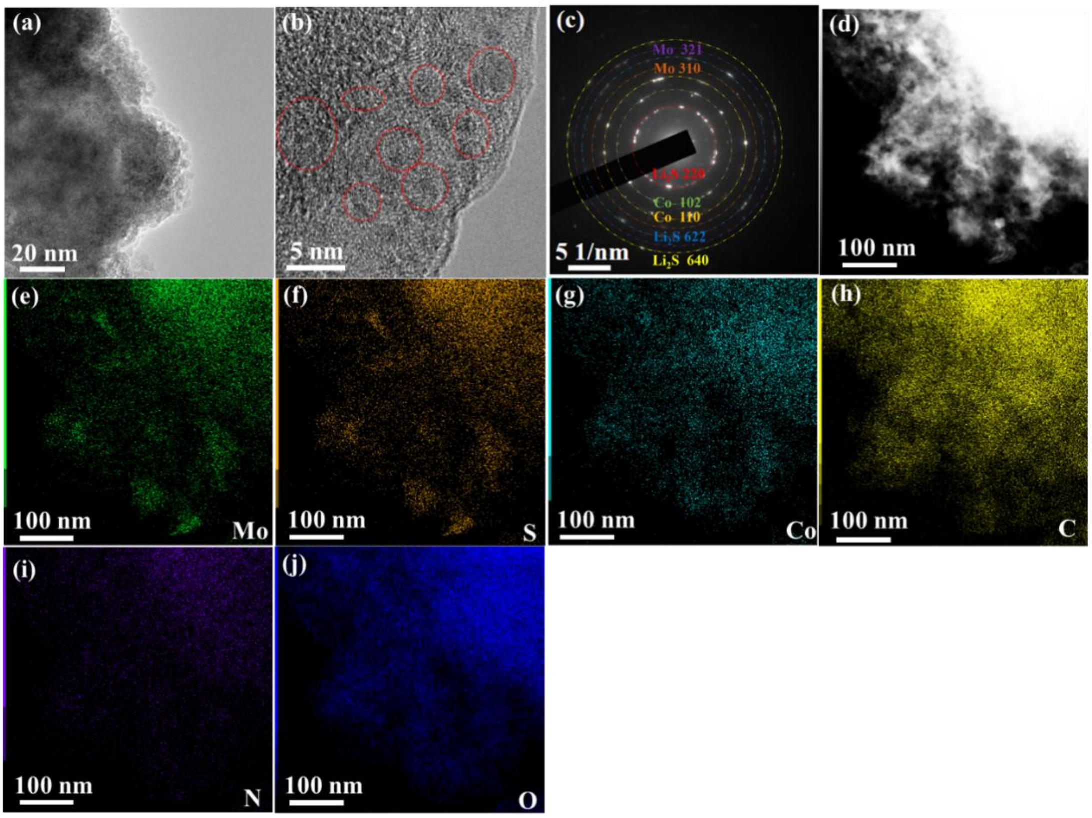


**Figure S24.** *Ex-situ* TEM characterizations of Co-FLMoS$_2$/NOC electrodes after discharging to 0.01 V. (a,b) TEM images, (c) corresponding SAED, (d) HAADF and corresponding EDS mapping images of Mo (e), S (f), Co (g), C (h), N (i), and O (j).

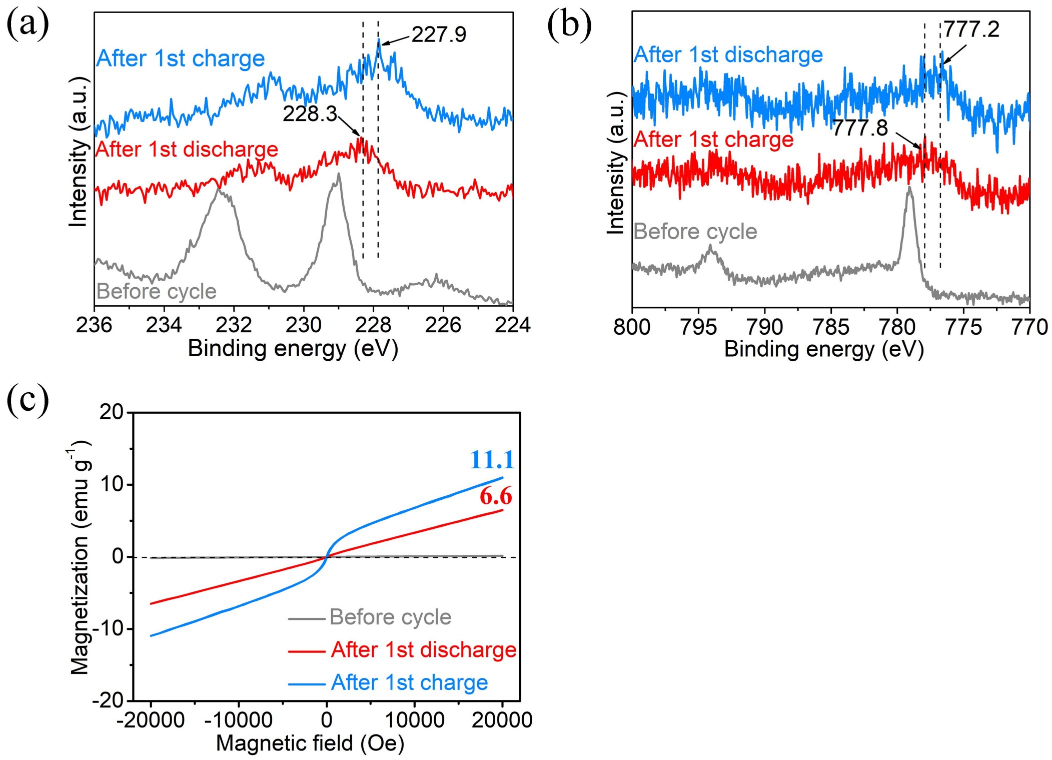


**Figure S25.** *Ex-situ* (a) Mo 3d XPS, (b) Co 2p XPS, and (c) magnetic hysteresis loops of Co-FLMo$S_2$/NOC electrodes after discharging to 0.01 V and charging to 3 V.

As can be seen in Figures S25a, b, the characteristic peaks of Mo 3d and Co 2p shift remarkably to lower binding energy (228.3 eV, $Mo^0$; 777.8 eV, $Co^0$) after discharging to 0.01 V, suggesting the formation of metal Mo and Co. Besides, a further negative shift of these peaks is detected (227.9 eV, $Mo^0$; 777.2 eV, $Co^0$), which arises from the delithiation from the Mo and Co surface. The pristine electrode shows a magnetization of about 0 emu $g^{-1}$ (Figure S25c), while an increased magnetization of 6.6 emu $g^{-1}$ is obtained after discharging to 0.01 V, which is addressed to the formation of superparamagnetic Mo and Co nanoparticles. Clearly, the magnetization increases to 11.1 emu $g^{-1}$ after charging to 3 V because of the delithiation from the Mo and Co surface. Consequently, the above results efficiently evidence the Li ions are stored on the Mo and Co surface to construct the space charge zone.

**Table S8** Electrochemical performances of $MoS_2$-based materials for LIBs anodes in open reports. $C_C$-final charge capacity (mAh $g^{-1}$), $C_R$-capacity retention (%), $M_L$-mass loading (mg $cm^{-2}$), J-current density (A $g^{-1}$), $N_C$-cycle number, NA-not available.

| Samples | $C_C$ | $C_R$ | $M_L$ | J | $N_C$ | References |
|---|---|---|---|---|---|---|
| **Co-SL$MoS_2$/NOC** | **1596.2** | **105.0** | **1.2** | **0.1** | **100** | **This work** |
| **Co-SL$MoS_2$/NOC** | **1372.2** | **98.9** | **1.2** | **1** | **1000** | **This work** |
| **Co-SL$MoS_2$/NOC** | **1084.3** | **90.1** | **1.2** | **5** | **3000** | **This work** |
| $TiO_2$@C@$MoS_2$ | 1150.0 | NA | 0.5 | 0.1 | NA | Adv. Energy Mater. **2018**, *8*, 1703155 |
| $TiO_2$@C@$MoS_2$ | 720 | 90 | 2 | 1 | 1500 | Adv. Energy Mater. **2018**, *8*, 1703155 |
| $TiO_2$@C@$MoS_2$ | 379.0 | NA | 0.5 | 5 | NA | Adv. Energy Mater. **2018**, *8*, 1703155 |
| N-GRs/$MoS_2$ | 1151 | NA | 1 | 0.1 | NA | Adv. Funct. Mater. **2018**, *28*, 1803690 |
| N-GRs/$MoS_2$ | 547 | 92.6 | 1 | 2 | 600 | Adv. Funct. Mater. **2018**, *28*, 1803690 |
| N-GRs/$MoS_2$ | 499.3 | NA | 1 | 8 | NA | Adv. Funct. Mater. **2018**, *28*, 1803690 |
| $MoS_2$/SnS | 988 | 100% | NA | 0.2 | 200 | Adv. Sci. **2018**, *5*, 1800241 |
| $MoS_2$/SnS | 634 | 53 | NA | 5 | 1000 | Adv. Sci. **2018**, *5*, 1800241 |
| $MoS_2$/SnS | 745 | NA | NA | 10 | NA | Adv. Sci. **2018**, *5*, 1800241 |
| PCN@$MoS_2$@C | 1052.5 | 88% | 1.5 | 0.1 | 200 | Chem. Eng. J. **2021**, *416*, 129094 |
| PCN@$MoS_2$@C | 609 | NA | 1.5 | 2 | NA | Chem. Eng. J. **2021**, *416*, 129094 |

| | | | | | | |
|---|---|---|---|---|---|---|
| $MoS_2$/NC-PNR | 800 | ~96.4 | 0.7 | 0.5 | 150 | Chem. Eng. J. **2021**, *408*, 127269 |
| $MoS_2$/NC-PNR | 520 | NA | 0.7 | 2 | 700 | Chem. Eng. J. **2021**, *408*, 127269 |
| $MoS_2$/NC-PNR | 443 | NA | 0.7 | 10 | NA | Chem. Eng. J. **2021**, *408*, 127269 |
| $MoS_2$/ $Mo_2TiC_2T_x$ | 509 | 91.88 | NA | 0.1 | 100 | Angew. Chem., Int. Ed. Engl. **2020**, *59*, 14621 |
| $MoS_2$/ $Mo_2TiC_2T_x$ | 182 | NA | NA | 2 | NA | Angew. Chem., Int. Ed. Engl. **2020**, *59*, 14621 |
| CNT@$MoS_2$@C | 905 | NA | 1.00 | 1 | 500 | Nano Energy **2019**, *65*, 104061 |
| RGO/$MoS_2$ | 892 | 93.89 | 0.41 | 2 | 400 | J. Mater. Chem. A **2019**, *7*, 7553 |
| RGO/$MoS_2$ | 723 | NA | 0.41 | 10 | NA | J. Mater. Chem. A **2019**, *7*, 7553 |

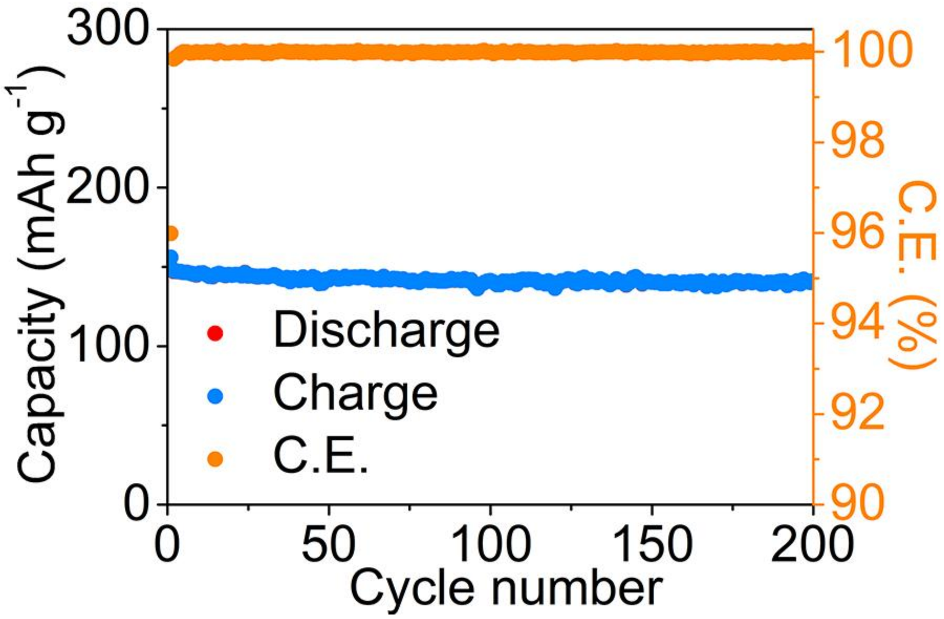


**Figure S26.** The cycling performances of the full cell at 1 C.

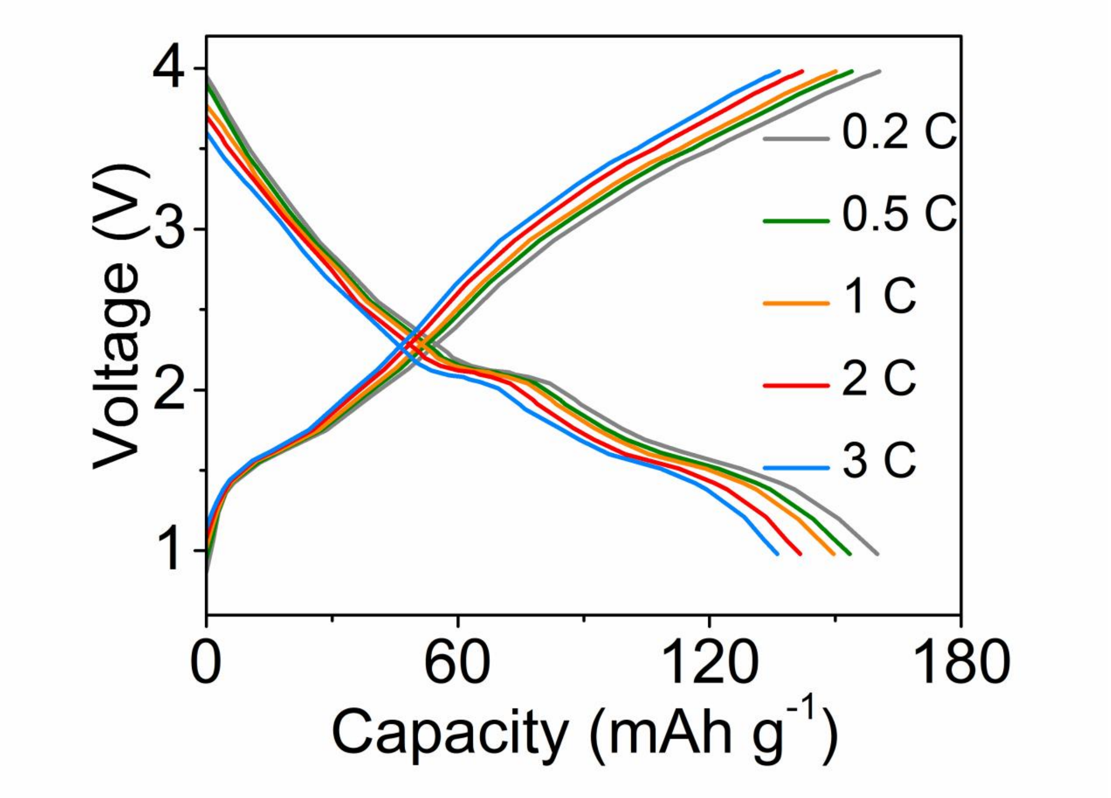


**Figure S27.** Rate capability of the full cell under different current densities.

**Table S9** Electrochemical performances of $MoS_2$-based anode materials in LIBs full cell. $C_C$-charge capacity (mAh $g^{-1}$), $C_R$-capacity retention (%), $M_L$-mass loading (mg $cm^{-2}$), J-current density (A $g^{-1}$, based on cathode), $N_C$-cycle number, NA-not available.

| Samples | $C_R$ | $M_L$ | J | $N_C$ | References |
|---|---|---|---|---|---|
| **Co-SLMoS2/NOC** | **97.2** | **1.8** | **0.017** | **100** | **This work** |
| **Co-SLMoS2/NOC** | **94.3** | **1.8** | **0.17** | **200** | **This work** |
| **Co-SLMoS2/NOC** | **81.9** | **1.8** | **0.51** | **500** | **This work** |
| $MnS-MoS_2$ | ~ 73 | 1.2 | 0.5 | 350 | Advanced Functional Materials 31.6 (2021): 2007132 |
| $SnS_2/MoS_2$/CFC | 63.5 | 1.5-2 mg $cm^{-2}$ | 0.15 | 50 | Chemical Engineering Journal 356 (2019): 483-491 |
| C-$MoS_2$ | 80 | ~ 1.0 | 0.19 | 50 | Chemical Engineering Journal 428 (2022): 131103 |
| N-GRs/$MoS_2$ | 86 | 1.0 | 0.1 | 200 | Chemical Engineering Journal 408 (2021): 127269 |
| $MoS_2$-NT | ~ 89 | 1.0 | 0.1 | 100 | Journal of Alloys and Compounds 907 (2022): 164499 |
| $MoO_2$@$MoS_2$/rGO | 81.6 | 1.5 | 0.2 | 40 | Electrochimica Acta 364 (2020): 136996 |

**Table S10** Electrochemical performances of $MoS_2$-based materials for SIB anodes in open reports. $C_C$-charge capacity (mAh $g^{-1}$), $C_R$-capacity retention (%), $M_L$-mass loading (mg $cm^{-2}$), J-current density (A $g^{-1}$), $N_C$-cycle number, NA-not available.

| Samples | $C_C$ | $C_R$ | $M_L$ | J | $N_C$ | References |
|---|---|---|---|---|---|---|
| **Co-SL$MoS_2$/NOC** | **861.5** | **100.3** | **1.2** | **0.1** | **100** | **This work** |
| **Co-SL$MoS_2$/NOC** | **521.9** | **86.0** | **1.2** | **5** | **3000** | **This work** |
| RGO/$MoS_2$ | 312 | 71.3 | 0.41 | 1 | 600 | *Nature* **2016**, *536*, 159 |
| $Cu_2S$@carbon@$MoS_2$ | 430 | NA | NA | 0.05 | NA | Angew. Chem., Int. Ed. **2020**, *59*, 7178 |
| $Cu_2S$@carbon@$MoS_2$ | 297 | NA | NA | 3 | NA | Angew. Chem., Int. Ed. **2020**, *59*, 7178 |
| $Cu_2S$@carbon@$MoS_2$ | ~280 | 80.4 | NA | 0.3 | 200 | Angew. Chem., Int. Ed. **2020**, *59*, 7178 |
| ANDC/$MoS_2$ | 496 | 94.5 | 1.3 | 1 | 1000 | ACS Nano **2021**, *15*, 13814 |
| ANDC/$MoS_2$ | 336 | 72.4 | 1.3 | 5 | 300 | ACS Nano **2021**, *15*, 13814 |
| $Nb_2CT_x$@$MoS_2$@C | 530 | 87.0 | ~1.1 | 0.1 | 200 | ACS Nano **2021**, *15*, 7439 |
| $Nb_2CT_x$@$MoS_2$@C | 403 | 80 | ~1.1 | 1 | 2000 | ACS Nano **2021**, *15*, 7439 |
| $Nb_2CT_x$@$MoS_2$@C | 340 | NA | ~1.1 | 20 | NA | ACS Nano **2021**, *15*, 7439 |
| N-$MoS_2$/C@SiOC | 540.7 | 127.9 | 1.5 | 0.1 | 200 | ACS Nano **2021**, *15*, 7409 |
| N-$MoS_2$/C@SiOC | 295.7 | NA | 1.5 | 10 | NA | ACS Nano **2021**, *15*, 7409 |
| N-$MoS_2$/C | 401 | 77.9 | 1 | 0.13 | 200 | Chem. Eng. J. **2020**, *387*, 124144 |

| | | | | | | |
|---|---|---|---|---|---|---|
| N-$MoS_2$/C | 388 | NA | 1 | 6.7 | NA | Chem. Eng. J. **2020**, *387*, 124144 |
| $MoS_2$/C | 419.5 | 91.5 | 1.2 | 0.067 | 100 | J. Mater. Chem. A **2019**, *7*, 4804 |
| $MoS_2$/C | 187.9 | NA | 1.2 | 3.35 | NA | J. Mater. Chem. A **2019**, *7*, 4804 |
| M-c $MoS_2$ | 401 | 89.1 | NA | 0.2 | 150 | Nano Energy **2018**, *51*, 546 |

**Table S11** Electrochemical performances of $MoS_2$-based materials for PIB anodes in open reports. $C_C$-charge capacity (mAh $g^{-1}$), $C_R$-capacity retention (%), $M_L$-mass loading (mg $cm^{-2}$), J-current density (A $g^{-1}$), $N_C$-cycle number, NA-not available.

| Samples | $C_C$ | $C_R$ | $M_L$ | J | $N_C$ | References |
|---|---|---|---|---|---|---|
| **Co-SL$MoS_2$/NOC** | **593.9** | **96.0** | **1.2** | **0.1** | **100** | **This work** |
| **Co-SL$MoS_2$/NOC** | **339.8** | **79.1** | **1.2** | **5** | **3000** | **This work** |
| $MoS_2$/N, P-rGO | 462.7 | 106.4 | 1.2 | 0.1 | 200 | Nano Res. **2021**, *14*, 3523 |
| 1T-$MoS_2$/graphene | 511 | 101.2 | 1 | 0.1 | 100 | Adv. Funct. Mater. **2020**, *30*, 2001484 |
| 1T-$MoS_2$/graphene | 246 | 75.2 | 1 | 1 | 800 | Adv. Funct. Mater. **2020**, *30*, 2001484 |
| $Fe_7S_8$/C@d-$MoS_2$ | ~260 | ~74.3 | 0.5 | 4 | 500 | ACS Nano **2020**, *14*, 16046 |
| $Fe_7S_8$/C@d-$MoS_2$ | 318 | NA | 0.5 | 5 | NA | ACS Nano **2020**, *14*, 16046 |
| exp-$MoS_2$ | 510 | 98.1 | 1 | 0.1 | 100 | Nano Res. **2020**, *13*, 225 |
| exp-$MoS_2$ | 310 | NA | 1 | 1 | NA | Nano Res. **2020**, *13*, 225 |
| $MoS_2$@HPCS | 126.2 | 60 | NA | 1 | 500 | ACS Appl. Mater. Interfaces **2020**, *12*, 1232 |
| $MoS_2$@HPCS | 93.1 | NA | NA | 2 | NA | ACS Appl. Mater. Interfaces **2020**, *12*, 1232 |
| $MoS_2$@$MoO_2$@Fe @CN | ~220 | 95 | NA | 0.1 | 100 | J. Mater. Chem. A **2019**, *7*, 26818 |
| $MoS_2$@$MoO_2$@Fe @CN | ~145 | 91 | NA | 0.5 | 500 | J. Mater. Chem. A **2019**, *7*, 26818 |

**References**


[1] Kresse G, Furthmüller J. *Comp. Mater. Sci.* **1996,** *6,* 15-50.

[2] Kresse G, Furthmüller J. *Phys. Rev. B.* **1996,** *54,* 11169-11186.

[3] Blöchl PE. *Phys. Rev. B.* **1994,** *50,* 17953-17979.

[4] Perdew JP, Burke K, Ernzerhof M. *Phys. Rev. Lett.* **1997,** *7*8, 1396-1396.

[5] Grimme S, Antony J, Ehrlich S. *J. Chem. Phys.* **2010,** *132,* 154104.

[6] C. Chen, X. Xie, B. Anasori, A. Sarycheva, T. Makaryan, M. Zhao, P. Urbankowski, L. Miao, J. Jiang, Y. Gogotsi, *Angew. Chem., Int. Ed. Engl.* **2018**, *57*, 1846.

[7] Z. Zhang, H. Zhao, Y. Teng, X. Chang, Q. Xia, Z. Li, J. Fang, Z. Du, K. Swierczek, *Adv. Energy Mater.* **2018**, *8*, 1700174.
